# Infection by a Giant Virus (AaV) Induces Widespread Physiological Reprogramming in *Aureococcus anophagefferens* CCMP1984 *–* A Harmful Bloom Algae

**DOI:** 10.3389/fmicb.2018.00752

**Published:** 2018-04-19

**Authors:** Mohammad Moniruzzaman, Eric R. Gann, Steven W. Wilhelm

**Affiliations:** ^1^Department of Microbiology, The University of Tennessee, Knoxville, Knoxville, TN, United States; ^2^Monterey Bay Aquarium Research Institute (MBARI), Moss Landing, CA, United States

**Keywords:** host–virus interaction, transcriptional profiles, *Mimiviridae*, algal viruses, brown tide

## Abstract

While viruses with distinct phylogenetic origins and different nucleic acid types can infect and lyse eukaryotic phytoplankton, “giant” dsDNA viruses have been found to be associated with important ecological processes, including the collapse of algal blooms. However, the molecular aspects of giant virus–host interactions remain largely unknown. Aureococcus anophagefferens virus (AaV), a giant virus in the *Mimiviridae* clade, is known to play a critical role in regulating the fate of brown tide blooms caused by the pelagophyte *Aureococcus anophagefferens.* To understand the physiological response of *A. anophagefferens* CCMP1984 upon AaV infection, we studied the transcriptomic landscape of this host–virus pair over an entire infection cycle using a RNA-sequencing approach. A massive transcriptional response of the host was evident as early as 5 min post-infection, with modulation of specific processes likely related to both host defense mechanism(s) and viral takeover of the cell. Infected *Aureococcus* showed a relative suppression of host-cell transcripts associated with photosynthesis, cytoskeleton formation, fatty acid, and carbohydrate biosynthesis. In contrast, host cell processes related to protein synthesis, polyamine biosynthesis, cellular respiration, transcription, and RNA processing were overrepresented compared to the healthy cultures at different stages of the infection cycle. A large number of redox active host-selenoproteins were overexpressed, which suggested that viral replication and assembly progresses in a highly oxidative environment. The majority (99.2%) of annotated AaV genes were expressed at some point during the infection cycle and demonstrated a clear temporal–expression pattern and an increasing relative expression for the majority of the genes through the time course. We detected a putative early promoter motif for AaV, which was highly similar to the early promoter elements of two other *Mimiviridae* members, indicating some degree of evolutionary conservation of gene regulation within this clade. This large-scale transcriptome study provides insights into the *Aureococcus* cells infected by a giant virus and establishes a foundation to test hypotheses regarding metabolic and regulatory processes critical for AaV and other *Mimiviridae* members.

## Introduction

Viruses are thought to lyse cells and release cellular organic and inorganic nutrients that either become available for microbial growth or are exported to the deep ocean (Wilhelm and Suttle, 1999). With an estimated 10^31^ virus particles in the sea (Angly et al., 2005), the geographical scale and impact of these processes are enormous – viral activity can turn over an estimate of 150 gigatons of carbon per year (Suttle, 2007). To accomplish this, it has been historically thought that viruses encode a minimal amount of genomic information that instructs host cells to produce new virus particles. Using almost entirely the host machineries, hundreds of virus particles can be produced from one host cell. As an example, Hepatitis B virus encodes only four overlapping genes in a 3.2-kb genome (Liang, 2009), whereas several *Picornavirales* members, which are widespread in the ocean, only code for one or two proteins (Lang et al., 2009).

This paradigm has been challenged by discovery of “giant” eukaryotic viruses – viruses that rival bacterial cells in terms of their physical size and genomic content (Raoult et al., 2004; Moniruzzaman et al., 2014; Wilhelm et al., 2016, 2017). Phylogenetic analyses of members of this group (known collectively as nucleocytoplasmic large DNA viruses, NCLDVs) (Iyer et al., 2001) have revealed that a major portion of the genomic content of these giant viruses has been acquired from the eukaryotic hosts and other sources through horizontal gene transfer (HGT), some of which are passed vertically through the course of viral evolution (Filee et al., 2007; Koonin and Yutin, 2010; Moniruzzaman et al., 2014). This genomic complement renders these viruses more autonomous from the host cell, empowering them to control individual processes in the complex eukaryotic cells and produce virus-specific macromolecules (Wilson et al., 2005; Claverie and Abergel, 2010).

Giant viruses are thought to play important roles in constraining photosynthetic protists in both marine and freshwater ecosystems (Short, 2012; Moniruzzaman et al., 2017). And while there is growing information regarding large virus diversity, seasonality, and roles in host dynamics, there is a dearth of information regarding the molecular underpinnings of conversion of a healthy host cell into a virus producing “machine” (*aka* the “virocell”) (Forterre, 2011). For example, molecular information is necessary to understand how viral infection can select for resistance in hosts, or shape the macromolecules released during cell lysis into surrounding waters. Indeed, given that infection is an ongoing and prevalent process in the oceans, a significant amount of the particulate chemistry that oceanographers measure may be due to infection phenotypes. Understanding the molecular aspects of giant virus infections can also reveal important markers of infection that can be used to track and differentiate infected cells from healthy cells *in situ*.

Giant viruses infecting eukaryotic algae are functionally diverse, although they do share a few core proteins (Yutin et al., 2009). As a consequence, significant differences in the molecular basis of interactions can be expected between different eukaryotic host–virus pairs, although not much is known in this regard. High-throughput techniques, like transcriptomics and/or metabolomics, have only been focused on a few ecologically relevant host–virus systems. From the work that exists we know that giant viruses genes, like those from Mimivirus and *Paramecium bursaria* Chlorella virus-1 (PBCV-1), are expressed quickly upon infection: PBCV-1 gene transcripts have been detected within 7 min of infection (Blanc et al., 2014). These viruses are also known to capture genes through HGT from their hosts and diverse sources, although function of these genes (and even if they are transcribed) remains largely unknown. Critical insights have been obtained regarding the modulation of cellular processes of *Emiliania huxleyi –* the most abundant coccolithophore alga in the world’s ocean – upon infection by large Emiliania huxleyi virus (EhV) (Vardi et al., 2012). This includes virus-mediated regulation of the host’s lipid biosynthesis resulting in programmed cell death and modulation of the host redox state during infection (Vardi et al., 2009; Rosenwasser et al., 2016). In Mimivirus, elaborate virus factories – cytoplasmic sites for virus replication and assembly – have been detected. Despite these important discoveries, a significant knowledge gap exists regarding the physiological response of a host to a giant virus infection.

*Aureococcus anophagefferens* is a bloom forming pelagophyte which causes recurrent brown tides along the east coast (Gobler et al., 2005). A giant virus (AaV) was isolated during a brown tide event and shown to infect and lyse *Aureococcus* in culture (Rowe et al., 2008). A subsequent genomic study revealed the “chimeric” nature of AaV; which picked up large number of genes from diverse cellular sources (Moniruzzaman et al., 2014), while statistical analysis of metatranscriptomic data from a brown tide bloom demonstrated active infection of *Aureococcus* by AaV during the peak of the bloom (Moniruzzaman et al., 2017). In addition, AaV is one of the few algae infecting viruses in the *Mimiviridae*, a clade of giant viruses that infect both photosynthetic and heterotrophic protists (Moniruzzaman et al., 2014). The availability of genome sequences for both AaV and its host (Gobler et al., 2011; Moniruzzaman et al., 2014) and recurrent brown tide blooms (Gastrich et al., 2004; Gobler et al., 2007) makes this host–virus pair an interesting model system. No information is available on the progressive changes in the molecular processes of *Aureococcus* cells upon infection, which might provide critical insights on the metabolic pathways and cellular components that can impact virus production. Moreover, the possible roles and activity of the large number of xenologs that AaV has acquired from its host, other organisms, and its NCLDV ancestor remain to be elucidated.

In this study, we employed transcriptomics to resolve the molecular response of *A. anophagefferens* to infection by AaV. Our experimental design examined the transcriptomic landscape throughout the AaV infection cycle to capture the host cellular response and viral transcriptional landscape. This study also provides insight into the molecular interaction between a giant algal virus in the *Mimiviridae* clade and its host.

## Materials and Methods

### Experimental Setup

*Aureococcus anophagefferens* CCMP 1984 was maintained in modified L-1 medium (Hallegraeff et al., 2003) at an irradiation level of 100 μmol photons m^-2^ s^-1^ and a temperature of 19°C for a 14:10 (h) light–dark cycle. Prior to the experiment, *Aureococcus* cultures were grown to a mid-log phase concentration of ∼1.95 × 10^6^ cells/ml. Five biological replicates (2.0 l) of *Aureococcus* cultures at a concentration of 7.5 × 10^5^ cells/ml were started within 2 h of the onset of the light cycle. The cultures were inoculated with AaV at a particle multiplicity of infection (pMOI) of ∼18. pMOI of 18 was chosen because it led to ∼98% reduction of cell numbers 48 h post-infection in assays conducted in-house. Due to the absence of a plaque assay to determine infectious units for AaV, we defined pMOI as total virus particles (not plaque forming units) counted using fluorescence microscopy for our experiment. For each biological replicate, control cultures were inoculated with the same volume of a heat-killed viral lysate. The heat-killed lysate was generated by exposure to microwaves (1000 Watts, 120 Volts) (Keller et al., 1988) for 5 min. Molecular aspects that define the different stages (early, intermediate, and late) of AaV infection are largely unknown. However, it is known that AaV has an approximately 24 h infection cycle (Brown and Bidle, 2014). Therefore, samples for sequencing were collected at 5 min, 30 min, 1 h, 6 h, 12 h, and 21 h after inoculation to capture a range of infection states before lysis (Brown and Bidle, 2014). Specifically, the first three time points were targeted to capture the cellular and viral gene expression changes that unfold within an hour of the infection, whereas the last time point was picked to investigate the events prior to cell lysis. In addition, the intermediate time points (6 and 12 h) were included to investigate how gene expression pattern changed as the cycle progressed from initial infection toward cell lysis. For RNA extraction, 250 ml subsamples were filtered through 0.8-μM pore-size ATTP filters (EMD Millipore, Darmstadt, Germany) to trap the algal cells. The filters were immediately flash frozen in liquid nitrogen prior to storage at -80°C. Unfiltered samples (for cell enumeration) and samples passed through 0.45-μm polyvinylidene fluoride syringe filters (Merck, Darmstadt, Germany) for free virus count were preserved in 0.5% glutaraldehyde at -80°C from each sample at each time point.

### Cell and Free Virus Density Estimates

*Aureococcus* cells were enumerated using a GUAVA-HT6 flow cytometer (EMD Millipore, Darmstadt, Germany) gated on the red chlorophyll fluorescence. Cell numbers were estimated from running the flow cytometer for 80 s or 5000 events per sample. Free virus particle densities from each time point was determined following Ortmann and Suttle (2009). Samples were thawed at room temperature and diluted 100-fold using L-1 medium prior to counting. The diluted samples were collected on 25-mm diameter Whatman Anodisc (Sigma-Aldrich, St. Louis, MO, United States) inorganic membrane filters having a nominal pore-size of 0.02 μm. The filters were allowed to air-dry for 15 min following incubation with 15 μl of 4000× diluted SYBER Green (Lonza, Rockland, ME, United States). The filters were then fixed using an anti-fade solution (50:50 PBS/glycerol and 0.1% *p*-phenylenediamine) (Noble and Fuhrman, 1998). Slides were observed through a Leica DM5500 B microscope at 1000× magnification with a L5 filter cube (excitation filter: 480/40, suppression filter: BP 527/30) (Leica Microsystems CMS GmbH, Hesse, Germany). For each sample, 20 random fields (1 μm by 1 μm) or 200 particles were enumerated and averaged. The following formula was used to estimate the VLPs/ml in each sample:

$$\begin{array}{c} VLPs/ml=V_{f}*\frac{A_{a}}{A_{g}*V_{f}}*D \end{array}$$

where *V*_f_ average virus count/field, *A*_a_ total filterable area of Anodisc (excluding the O-ring), *A*_g_ area of eyepiece grid, *V*_f_ volume filtered (ml), and *D* dilution factor.

### RNA Extraction and Sequencing

Three biological replicate experiments were used for RNA extraction and analyses at each time point. RNA was extracted with MO BIO PowerWater RNA Isolation Kit [MO BIO Laboratories (now QIAGEN), Carlsbad, CA, United States] following a 2-min bead beating step using Lysing Matrix E 2 mL tubes (MP Biomedicals, Santa Ana, CA, United States). The manufacturer’s protocol was followed with slight modification: specifically, the DNAse treatment step was performed twice to ensure sufficient purity of the RNA. RNA was quantified using a Nanodrop ND-1000 Spectrophotometer (Thermo Scientific, Waltham, MA, United States) and RNA integrity was checked with an Agilent Bioanalyzer 2100 (Agilent Technologies, Santa Clara, CA, United States). Extracted RNA was processed and sequenced at the Hudson Alpha Genomic Services Lab (Huntsville, AL, United States). RNA samples were poly-A selected to enrich for mRNA and deplete ribosomal RNA transcripts. Samples were sequenced using an Illumina^®^ NextSeq^®^ sequencer targeting approximately 25 million single-end reads per sample and a 76-bp read length. Standard protocols by Illumina^®^ were followed for library preparation, poly-dT bead selection, and sequencing. Sequence data have been deposited in the NCBI short-read archive under bioproject PRJNA432024.

### Bioinformatics and Statistical Analysis

Sequencing reads were initially trimmed in CLC Genomics Workbench 9.0 (Qiagen, Hilden, Germany). Reads with a quality score cut-off of ≤0.03, or with ambiguous bases (“N”s), were discarded. Reads passing quality control were mapped to the *Aureococcus* (NCBI Accession No. ACJI00000000) and AaV genome sequence (NCBI Accession No. NC_024697) with stringent mapping criteria (95% similarity, 70% length matching). Differential expression of genes in the virus-treated samples compared to the controls was determined at each time point using edgeR (Robinson et al., 2010) program implemented in the CLC Genomics Workbench 9.0. *P*-values were adjusted for false discovery rate (FDR) using Benjamini–Hochberg (BH) procedure (Benjamini and Hochberg, 1995). Heatmaps representing data from these analyses (**Figures 5, 6** and Supplementary Figures 7, 9, 10) were constructed using statistical computing environment R (R Core Team, 2013). The number of reads mapped to each AaV gene was rarefied by library size. Values from biological replicates at each time point were averaged prior to hierarchical clustering of the viral gene expression. **Figure 2A** summarizing this data was constructed using BRIG software (Alikhan et al., 2011). The putative early promoter motif of AaV was detected using MEME (Bailey et al., 2009) in discriminative mode. The *E*-values associated with the discovered motifs are a conservative estimate of the number of motifs having equal or higher log-likelihood ratio if the target sequences were randomly generated (Bailey and Elkan, 1994) given a background model.

Functional enrichment within the framework of Gene Ontology (GO) terms (positive or negative fold changes) was determined using BiNGO (Maere et al., 2005). GO enrichment for differentially expressed genes is complicated by at an arbitrary fold-change cut-off imposed prior to the enrichment analysis, which excludes the genes with fold-change values even marginally similar to that cut-off. To partially alleviate this problem, we ran the enrichment analysis on gene sets selected using two absolute fold-change cut-offs: >1.5 and >1.3. Using both these cut-offs recovered mostly same GO processes; however, some of the processes were missed by each of the individual approaches. Since our analysis is largely exploratory, results obtained from both cut-off were investigated for interesting GO processes. We report all the GO terms recovered by this approach in Supplementary Dataset 1. The up- or down-regulation of KEGG pathway-related gene expression was determined using *z*-test as implemented in “GAGE” R package (Luo et al., 2009). This analysis employed input from all the genes, irrespective of fold-change level or statistical significance, and looked for coordinated expression changes within a particular pathway. The resulting *P*-values for both the analyses were corrected for FDR using BH procedure (Benjamini and Hochberg, 1995). We considered a FDR-corrected *P*-value ≤ 0.1 to be significant for both GO and KEGG pathway enrichment. Heatmaps showing the presence or absence of significantly enriched GO processes (**Figure 4** and Supplementary Figure 6) were constructed in statistical computing environment R (R Core Team, 2013). nMDS and clustering analysis of the virus and host gene expression data and associated figure construction were done using the statistical program PRIMER v6.0 (Clarke, 1993).

## Results

### Cell Growth Dynamics and RNA-Seq Output

**FIGURE 1:**
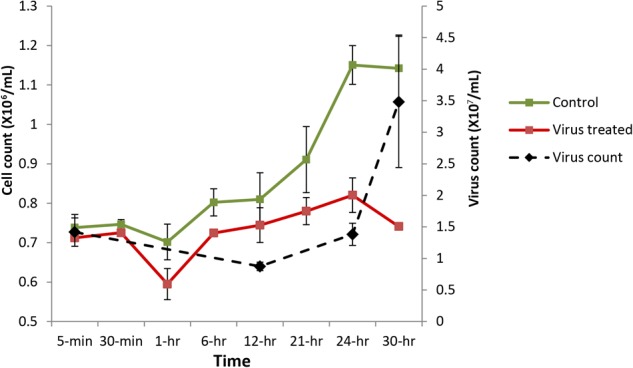
*Aureococcus* and AaV count over the course of infection. Cell counts are average of three biological replicates, while virus counts are average of two biological replicates. Green, cell count in control cultures; red, cell counts in infected cultures; and black, virus counts.

Cultures inoculated with heat-killed lysates displayed growth patterns similar to a healthy *Aureococcus* culture, reaching a cell density of approximately ∼1.1 × 10^6^ cells/ml by 24 h (**Figure 1** and Supplementary Figure 1). In contrast, the virus-infected cultures didn’t show any significant increase in cell density over the course of infection, indicating that a proportion of cells were infected during the first cycle of virus propagation. Consistent with previous studies, free-virus titer increased around 24 h after infection and steadily increased up to ∼3.5 × 10^7^ VLPs/ml by 30 h post-infection (**Figure 1**). Flow cytogram of infected culture shows distinct signature for cells going through lysis – with a subpopulation of cells having lower red fluorescence along with the population of uninfected cells (Supplementary Figure 2). Complete lysis of the culture usually takes 48–72 h during routine virus production in lab, encompassing two to three infection cycles, in agreement with the results reported previously (Rowe et al., 2008).

After quality trimming, between ∼18.2 and 29.4 million reads were obtained from each of the 36 samples. In the control samples, ∼80% of the reads could be mapped to the host nuclear (NCBI Accession No. ACJI00000000) (Gobler et al., 2011), chloroplast (Accession No. GQ231541) (Ong et al., 2010), and draft mitochondrial genome (scaffold 85 of *Aureococcus* genome project^1^) (Supplementary Table 1). Less than 1% reads mapped to chloroplast and mitochondrial genome across all samples (Supplementary Table 1). Such lower number of reads captured from organelles is possibly due to different dynamics of polyadenylation in the organelles compared to the nuclear genome. In eukaryotic nuclear transcripts, polyadenylation is an important step for mRNA maturation. However, polyadenylation of organelle and bacterial transcripts leads to their degradation (Chang and Tong, 2012). In the virus-treated samples, the proportion of virus transcripts steadily increased over time (Supplementary Table 1 and Supplementary Figure 3). *Aureococcus* culture used for this experiment was not axenic. About ∼20% of the reads from all the samples could not be aligned to the host or viral genomes, which likely originated from incomplete parts of the host genome, and/or polyadenylated transcripts from bacteria.

### Gene Expression Dynamics of AaV

**FIGURE 2:**
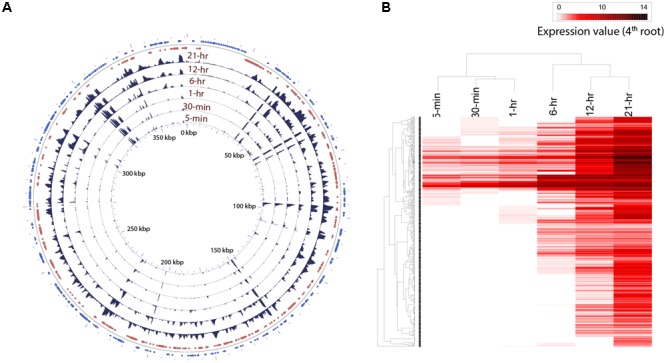
**(A)** Trend in expression of individual AaV genes over time. The read mapping from each time point was converted to coverage graphs with a 100 bp sliding window. Three replicates from each time points were averaged after rarefaction by library size. The two outermost rings represent the forward (blue) and reverse (red) coding sequences. **(B)** Heatmap showing temporal pattern of expression of clusters of AaV genes. The read mapping data over individual genes was hierarchically clustered after fourth root transformation.

Transcripts from 116 viral genes were present in the infected culture as early as 5 min post-infection (Supplementary Table 2 and Supplementary Figure 3). While only ∼0.007% of the reads could be mapped to viral genome from sequence libraries at 5 min, ∼15% of the reads originated from viral transcripts by the 21 h time point (**Figure 2A** and Supplementary Figure 3). We also observed a high coefficient of variation within replicates for the samples from the first three time points (Supplementary Figure 3). To resolve temporal patterns of virus gene expression, we performed a hierarchical clustering using the average number of rarefied reads per library that mapped to viral genes over the time course. Clear temporal patterns in gene expression were observed, with some genes expressed either immediately or within 1 h of infection, while reads from other genes appeared later into the infection (**Figure 2B**). Although the length of AaV infection cycle is known, data on the specific length of early or late infection stages are not available. Also, even though the first three samples were collected within an hour, there was a large interval between the third (1-h) and fourth (6-h) samples. So, it is possible that expression of any early genes within that interval could only be observed at 6-h time point. Therefore, we checked the genes expressed within the first four time points together for enrichment of possibly early promoter motifs on their upstream sequences. MEME (Bailey et al., 2009) was used in the discriminative mode to detect. A motif with the general pattern “[AT][AT][AT][TA]AAAAATGAT[ATG][AG][AC]AAA[AT]” was found to be enriched in the gene set expressed within 6 h with an *E*-value of 2.1e-151, compared to the genes expressed during the last two time points (Supplementary Figure 4A). This motif encompasses the octamer “AAAAATGA.” When we searched for the AaV-specific octamer motif, we found that 47.5% (127 genes) of the genes expressed within the 6 h of infection contain this motif with exact match on their upstream, while only 22% (24 genes) of the genes expressed during the last two time points harbored it in the upstream regions. A search for putative late promoter motif in the second set of sequence resulted in a statistically insignificant highly degenerate motif (*E*-value < 3.53e + 011) without any match to the previously reported late promoter motifs of giant viruses (Supplementary Figure 4B).

Read mapping to the AaV genome revealed that the expression of different genes had a large spatiotemporal variation (**Figure 2A**). Only three of the annotated genes from the AaV genome (AaV_004 and AaV_115 – hypothetical proteins and AaV_336 – a putative leucyl tRNA) were not detected as transcripts. The two terminal DUF285 domain-rich regions showed lower variation in expression values compared to other viral genes (**Figure 2A**). While relative expression of most of the viral genes varied across several orders of magnitude during the time course, expression of DUF285 regions stayed consistent within one order of magnitude. As a striking contrast, expression of major capsid protein was found to be dramatically high at 21 h – encompassing >50% of the virus-specific reads and ∼6% of the entire libraries at that time point.

Out of 384 genes in AaV (Moniruzzaman et al., 2014), only 137 have nucleocytoplasmic large DNA virus orthologous groups (NCVOG) (Yutin et al., 2009) and/or cluster of orthologous groups (COG) (Tatusov et al., 2000) assignments, giving insights into their potential function (Supplementary Table 2). Based on the cluster analysis, we found seven of nine annotated viral-methyltransferases were expressed within 6 h of infection. Expression of three out of four genes with NCVOG category “virion structure and morphogenesis” (AaV_165, 247 and 290) was detected at 12 or 21 h of infection – consistent with previous observations that genes involved in the production of virus structural components are expressed late during infection (Fischer et al., 2010; Legendre et al., 2010) (Supplementary Table 2). The exception was major capsid protein (AaV_096), a major structural component of the virus, which was found to be expressed immediately (5 min) after infection. Three ubiquitin ligases (AaV_228, 235, and 298) and two proteases (AaV_042, 066) were also found to be expressed 5 min post-infection, alongside a number of putative transcription factors (Supplementary Table 2). AaV has a number of genes unique among NCLDVs, which are putatively acquired by HGT from the host and other cellular organisms (Moniruzzaman et al., 2014). Among these, three carbohydrate metabolism genes (carbohydrate sulfotransferase: AaV_102, glucuronyl hydrolase: AaV_078, and pectate lyase: AaV_375) were expressed immediately after infection (Supplementary Table 2). However, the majority of HGT-acquired genes were found to be expressed only at or after 6 h (Supplementary Table 2). Most other genes with COG or NCVOG classifications did not show any “function-specific” temporal pattern (Supplementary Table 2).

### Global Transcriptional Remodeling of the Virus-Infected Host

**FIGURE 3:**
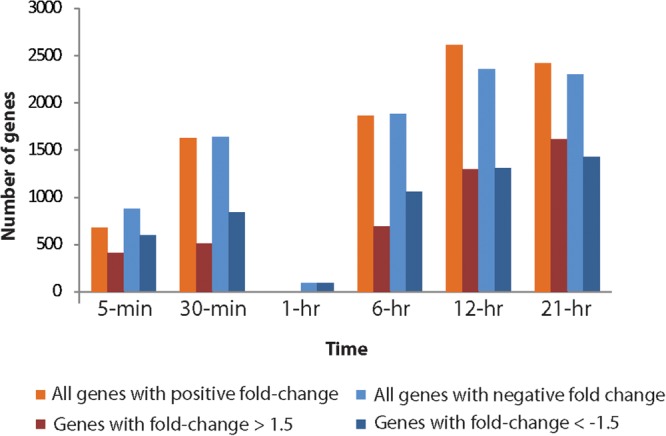
Total number of genes up- and down-regulated in the infected host across different time points compared to the healthy cultures. Number of up- or down- regulated genes are represented on the *Y*-axis.

Viral infection induced a dramatic and rapid reprogramming of host cell gene expression, which was reflected in the number of *Aureococcus* genes that were differentially expressed compared to the uninfected culture (**Figure 3**). Even at 5-min post-infection, we observed 13.4% of the 11,570 genes of *Aureococcus* were differentially expressed, with 412 genes having fold changes of >1.5, and 588 genes with a fold change less than -1.5 (FDR-corrected *p* < 0.05) (**Figure 3**). With exception of the 1-h time point, the number of genes over or underexpressed compared to control showed a tendency to increase over time, with the highest number of genes observed to be differentially expressed occurring at the 12 h time point (42.9%).

The number of differentially expressed host genes was dramatically reduced at 1-h compared to other time points: only 82 genes were found to be differentially expressed (**Figure 3**). All of these differentially expressed genes showed negative fold changes. Since the general trend of increasing number of differentially expressed genes over time did not apply to the 1-h time point, it presented an “anomaly” that invited further investigation. There were ∼9800 virus reads on average in the three virus-treated biological replicates from time point 1-h (Supplementary Figure 3), which is higher than the number of virus reads in 30-min samples and lower than that of 6-h samples. Thus, the number of total viral reads during 1-h time point fit the trend of increasing viral reads over time in the treated samples. An nMDS analysis coupled with a hierarchical clustering using Bray–Curtis similarity showed that control and infected samples from 1-h time point clustered together and showed >97.5% similarity between the replicates (Supplementary Figure 5). In contrast, corresponding control and treatment samples from other time points generally had a similarity below 95% and clustered according to treatments.

**FIGURE 4:**
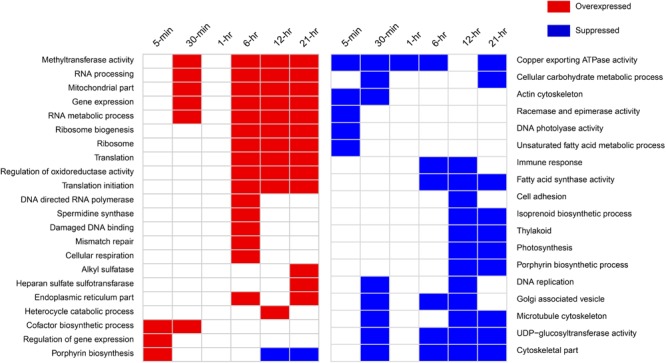
Notable GO categories overexpressed or suppressed in the host in virus-treated samples relative to controls over different time points. Overexpressed GO categories are those significantly enriched (FDR-corrected *p* < 0.1) in gene sets with >1.5-fold change, whereas suppressed GO categories are enriched in the gene sets with less than –1.5-fold change.

A large number of GO categories and KEGG pathways were differentially represented across time points, except for at 1-h (**Figure 4** and Supplementary Figure 6). Remarkably, several processes were up- or down-regulated almost immediately after infection. For example, overrepresentation of GO term “cofactor biosynthesis” and “regulation of gene expression” was found by 5-min post-infection, while actin and microtubule cytoskeleton, copper exporting ATPase, and racemase and epimerase activity-related GO processes were suppressed in the infected cells. Cytoskeleton-related GO terms were suppressed throughout the infection cycle. We found increased representation of ribosome, translation, and endoplasmic reticulum-related processes in the infected culture. Mitochondria and cellular respiration-related GO terms were also overrepresented. Notable processes that were suppressed in the infected culture were racemase and epimerase activity, unsaturated fatty acid metabolism, and Golgi-associated vesicle processes. The down-regulation of racemase and epimerase activity suggests a decrease in carbohydrate metabolism in the cell upon infection. Even though only a few genes were differentially expressed at 1-h, pathway analyses suggested increase in ribosome, butanoate, and sulfur metabolism-related gene expression at this time point (Supplementary Figure 6).

### DNA Mismatch Repair, “Immune Response,” and Polyamine Biosynthesis

Surprisingly, we detected a GO term “immune response” that was suppressed around 6- and 12-h post-infection (Supplementary Dataset 1). Upon closer inspection, the genes annotated by this function were found to be guanylate-binding proteins (GBPs). GBPs belong to interferon-gamma inducible GTPase superfamily that are widely known to promote defense against viruses and other intracellular pathogens in humans, mice, and other mammals (Vestal and Jeyaratnam, 2011). We determined that a number of GBP genes were down-regulated 30-min post-infection and onward (Supplementary Table 3). In addition, the phagosome pathway-related genes were also down-regulated immediately after infection, while genes involved in autophagy were down-regulated 6-h post-infection (**Figure 4**).

DNA mismatch repair (MMR) and damaged DNA-binding-related GO processes were found to be up-regulated around 6-h post-infection at transcriptional level (**Figure 4**). All of the genes annotated with these GO processes were found to encode proteins having MutS and MutL domains. Viral infection induced significant over-expression of a number of these genes within 5-min of infection (Supplementary Figure 7). These include a MutS homolog and a small MutS-related protein (smr). By 30 min, more of these genes showed significantly increased expression, which include a MutS2, MutL, and another smr homolog (Supplementary Figure 7). Around 6-h, all these genes remained highly expressed compared to control. Several genes were found to be down-regulated at 12 h but up-regulated at 21 h, indicating varying degree of regulatory controls acting upon them over the course of infection (Supplementary Figure 7).

Data exploration using GO analysis also revealed higher expression of spermidine synthase activity at 6 h post-infection compared to the control. Ornithine decarboxylase converts L-ornithine to putrescine, which is further converted to spermidine by spermidine synthase. *Aureococcus* homologs of ornithine decarboxylase and spermidine synthase were found to be up-regulated in the virus-infected culture from 6 h onward (Supplementary Figure 8). Additionally, a homolog of *N*-carbamoylputrescine amidase (Aurandraft_59241) was also found to be up-regulated during this time.

### Alteration of Photosynthesis and Photoprotection-Related Processes Upon Virus Infection

We observed significant down-regulation (FDR, *p* < 0.1) in 17 of the 62 light harvesting complex (LHC) genes in *Aureococcus* as early as 5 min after infection, indicating that the light harvesting capacity of the infected cells decreased compared to the healthy culture immediately upon infection (Supplementary Figure 8). The number of LHC genes showing negative fold-change compared to control increased through the infection time course – 41 genes at 30 min and 40 genes at 6 h had significantly reduced expression. By 12 and 21 h, most of the 62 LHC genes showed significant down-regulation, with the exception of 6 genes at 12 h and 2 genes at 21 h that showed overexpression (Supplementary Figure 9). Chloroplast genes encoding proteins for Photosystem I and II during the infection (*psaA, psaB, psaL, psbA, psbC*, and *psbD*) were down-regulated by 5 min post-infection (Supplementary Table 4). Taken together, the data indicate that transcription of genes crucial for photosynthesis decreased in the virus-infected cells throughout the light cycle. During the 12- and 21-h, photosynthesis and thylakoid-related GO processes were suppressed in the infected cells compared to the non-infected ones (**Figure 4**). This could indicate that photosynthesis-related genes peaked in expression during mid-night and pre-dawn in the healthy cultures, a phenomenon that has been observed in other algae such as *Ostreococcus tauri* (Monnier et al., 2010). In addition, isoprenoid biosynthesis genes were down-regulated around the same time. Expression of 3 of the 14 genes annotated with GO term “isoprenoid biosynthesis” was suppressed significantly at 5-min post-infection, while one was overexpressed (Supplementary Table 4). No significant change was detected for other isoprenoid biosynthesis genes by 5 min. The number of transcripts with reduced expression increased over time – eight genes were down-regulated by 30-min and 6-h of infection (Supplementary Table 5). This data indicated a decrease in isoprenoid biosynthesis-related transcripts in the virus-treated culture throughout the infection process.

**FIGURE 5:**
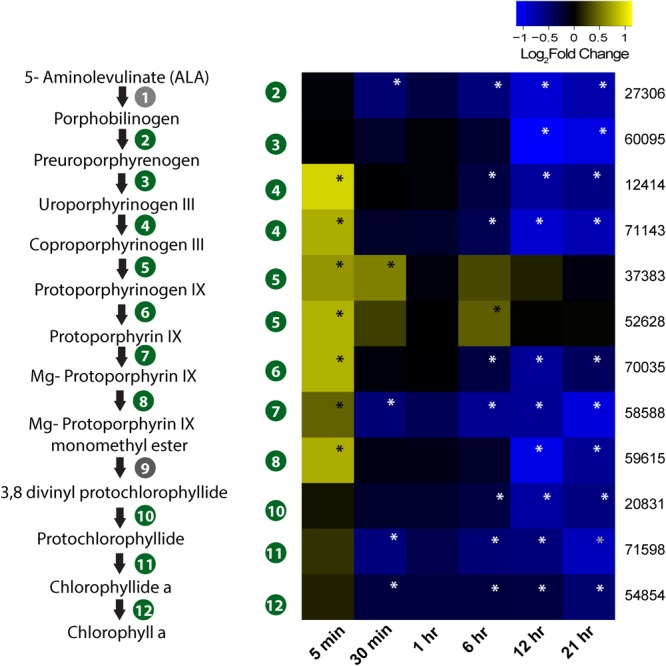
Expression profile of genes involved in porphyrin and chlorophyll biosynthesis in the infected culture. The pathway from ALA to chlorophyll *a* biosynthesis is also presented. Genes marked in gray circle (1 and 9) are not annotated in *Aureococcus*. Numbers given on the right side of the heatmap are JGI IDs. Annotated genes are as follows: 27306 – hydroxymethylbilane synthase/porphobilinogen deaminase; 60095 – uroporphyrinogen synthase; 12414 and 71143 – uroporphyrinogen decarboxylase; 37383 and 52628 – coproporphyrinogen III oxidase; 70035 – protoporphyrinogen oxidase chlorophyll precursor; 58588 – Mg chelatase ATPase; 59615 – mg protoporphyrin IX methyltransferase; 20831 – 3,8-divinyl protochlorophyllide a 8-vinyl reductase; 71598 – protochlorophyllide reductase; 54854 – chlorophyll synthase. Significant fold changes (FDR, *p* ≤ 0.1) are marked with asterisks.

The heme biosynthesis pathway, which leads to the production of photosynthetic pigments including chlorophyll a and other tetrapyrrolic pigments, is a crucial metabolic pathway in photosynthetic organisms (Obornik and Green, 2005). While genes involved in light harvesting, photosystem structure, and isoprenoid biosynthesis were generally under-expressed, we found porphyrin biosynthesis genes in this pathway to be overexpressed immediately after infection (**Figure 5**). All the genes involved in synthesis of the precursor of protoporphyrin-X were up-regulated immediately after infection, but were down-regulated by 30-min post-infection (**Figure 5**). In contrast, genes involved in chlorophyll *a* biosynthesis from protoporphyrin-X did not show significant up- or down-regulation at this time (**Figure 5**). Collectively, this data indicate that porphyrin derivatives accumulated early in the infected cell, possibly leading to photooxidative damage of different cellular components, including chloroplasts (Reinbothe et al., 1996). Additionally, genes involved in DNA photolyase activity was found to be down-regulated 5-min post-infection (**Figure 4**) – a process critical in repairing UV-mediated formation of pyrimidine dimers in DNA (Thiagarajan et al., 2011).

### Changes in Expression Associated With the Selenoproteome

**FIGURE 6:**
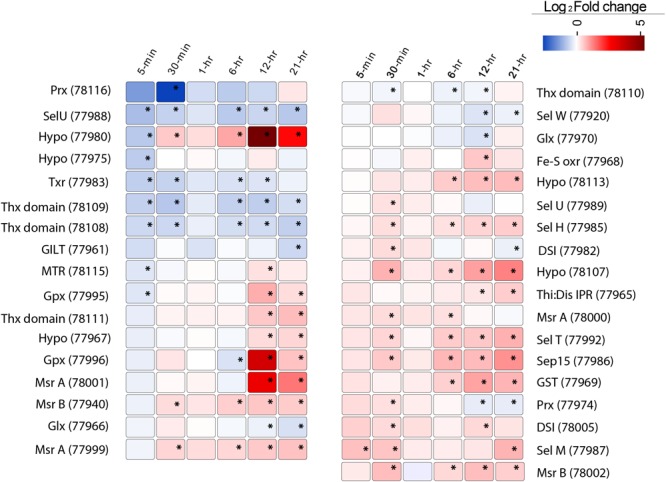
The expression pattern of 35 selenoproteins in *Aureococcus* showing significant fold change (FDR, *p* ≤ 0.1) in the virus infected culture at least at one time point. The significant fold changes are marked with asterisks. Abbreviations: MTR, methyltransferase; GILT, GILT superfamily protein; Thx domain, thioredoxin domain containing proteins; Thx red, thioredoxin reductase; Sel, selenoproteins; Hypo, hypothetical proteins; Prx, peroxiredoxin; Gpx, glutathione peroxidase; Glx, glutaredoxin; MSR, methionine sulfoxide reductases; DSI, protein disulfide isomerase; Sep15, selenoprotein 15; GST, glutathione-*S*-reductase; Fe-S oxr, Fe-S oxidoreductase.

*Aureococcus* has 59 predicted selenoproteins – the highest reported among all eukaryotes (Gobler et al., 2011). Out of these, 35 showed significant (FDR, *p* < 0.1) deregulation at least at one time point (**Figure 6**). While several selenoproteins genes were found to be under-expressed immediately post-infection, at 12- and 21-h a large number of selenoproteins genes showed increased expression relative to controls (**Figure 6**). We found *O*-phosphoseryl-tRNA (Sec) selenium transferase, a gene involved in producing selenocysteinyl-tRNA from L-seryl-tRNA (Sec) to be overexpressed (Supplementary Figure 10). Cystathionine beta-lyase and Selenocysteine (Sec) lyase, two genes involved in conversion of Sec into methionine and alanine, also showed increased expression compared to control (Supplementary Figure 10). Although no known selenium transporter has been characterized in *Aureococcus*, it is known that opportunistic transport of selenium using phosphate transporters might be common in plants, fungi, and algae (Lazard et al., 2010). *Aureococcus* has six annotated phosphate transporters. Among these, either three or four of the transporters showed significantly higher expression at 12- and 21-h, respectively, during infection compared to control culture (Supplementary Figure 11). Five of the overexpressed selenoprotein genes were methionine sulfoxide reductases (MSR) (**Figure 6**), genes involved in reversing the oxidation of methionine by reactive oxygen species (ROS), thereby repairing the oxidative damage in proteins (Moskovitz, 2005). Three copies of glutathione peroxidases (GPx) were also over-expressed compared to control, which are crucial in reducing H_2_O_2_ or organic hydroperoxides, thereby minimizing oxidative damage to cellular components (Ursini et al., 1995). Glutathione-*S*-transferase (GST), a selenoprotein gene with diverse function in cellular stress protection, was over-expressed during the last three time points. GSTs have diverse functions in the cell, including detoxification of electrophilic metabolites of xenobiotics into less reactive compounds by catalyzing their conjugation with glutathione (GSH) (Veal et al., 2002). GSTs are also known to participate in oxidative stress protection by conjugating GSH with secondary ROS molecules that are produced upon ROS reacting with cellular constituents (Danielson et al., 1987). In addition, some GSTs show GPx activity (Tan and Board, 1996). Sel U and Sel H, two selenoproteins involved in redox functions and Sep15, a transcript whose product is involved in protein folding in endoplasmic reticulum (Labunskyy et al., 2009) were overexpressed in the late stage of virus infection (**Figure 6**).

A number of genes encoding redox active proteins not incorporating selenium were also overexpressed during the last two time points. This includes a Cu–Zn superoxide dismutase (Aurandraft_59136), which dismutates superoxide anion ($O_{2}^{–}$), leading to production of H_2_O_2_. We detected overexpression of a dehydroascorbate reductase homolog (Aurandraft_67072), which is involved in recycling of ascorbate, during the last three time points. Ascorbate acts as a key antioxidant in the cell by directly neutralizing superoxide radicals, singlet oxygen, or hydroxyl radical (Noctor and Foyer, 1998).

## Discussion

### Transcriptional Landscape of AaV Infection

This study provides initial insight into the gene expression dynamics of an algal virus in the *Mimiviridae* clade (Santini et al., 2013). AaV has a 21–30 h infection cycle, with free virus production observable by 21 h post-infection, which steadily increases over time (Brown and Bidle, 2014). The almost immediate transcription of viral genes indicated a rapid modulation of host cellular processes directed toward transcribing the viral mRNAs (**Figure 2A** and Supplementary Figure 3). Fast transcription of viral genes was also observed in the *Chlorella*-infecting virus PBCV-1 transcriptome, where viral transcripts were detected 7 min post-infection (Blanc et al., 2014). How giant viruses accomplish this rapid transcription upon infection is an open question. It has been established that giant viruses like Mimivirus and *Cafeteria roenbergensis* virus incorporate numerous protein products within their capsids (Renesto et al., 2006; Fischer et al., 2014). Interestingly, Mimivirus particles package 12 transcription-related protein products, including all five DNA polymerase subunits and four transcription factors encoded by the viral genome (Renesto et al., 2006). This suggests that the fast transcription of Mimivirus genes could happen immediately after host cell entry. In addition, a qPCR approach could detect several Mimivirus transcripts from purified virus particles (Raoult et al., 2004), although DNA and RNA contamination of the particles cannot be completely ruled out. Also, RNA-seq analysis identified viral transcripts within 15 min of virus addition to *Acanthamoeba* culture (Legendre et al., 2010). However, this cannot definitively answer whether those transcripts were packaged in the particles or were transcribed immediately upon viral entry. It is possible that the appearance of viral transcript within 5 min of AaV infection resulted from the presence of mRNA and/or transcription associated enzymes within the virus particles. It has also been demonstrated that for Mimivirus (Legendre et al., 2010), *C. roenbergensis* virus (Fischer et al., 2010) and PBCV-1 (Blanc et al., 2014) almost all the genes are detected during the course of infection: AaV is not an exception. An interesting observation was the low level yet relatively stable expression of the terminal DUF285 domain containing genes compared to other viral genes, majority of which had increased expression level as time progressed. Terminally repetitive genes like these are a common feature of *Mimiviridae* members and are thought to be originated from massive gene duplication (Raoult et al., 2004; Fischer et al., 2010; Moniruzzaman et al., 2014). The regulatory mechanism behind the low expression dynamics of these genes is unknown – however, the role of methylation or binding of specific factors suppressing the expression of these genes can’t be ruled out. A highly conserved motif was found to be present upstream of these DUF285 genes (Moniruzzaman et al., 2014), which might be a candidate-binding site for certain regulatory factor(s).

Viral transcription increased over time, and at 21 h post-infection (just before the cell lysis) ∼15% of the reads came from viral transcripts. This is a low proportion of viral reads compared to what has been observed in Mimivirus (Legendre et al., 2010) and PBCV-1 (Blanc et al., 2014) infection transcriptomes. It appears that not all the *Aureococcus* cells may be infected during the first cycle, since it took the equivalent of two consecutive infection cycles (48 h) for a ∼98% reduction from the initial cell count. A study on EhV transcriptomics and metabolomics revealed that *E. huxleyi* cell counts remained relatively unchanged by 24 h of infection (Rosenwasser et al., 2014), similar to *Aureococcus* counts in this study. However, in that study ∼75–80% of the reads originated from EhV transcripts by 24 h. Why we saw only 15% AaV reads around the time of lytic burst remains an open question. This phenomenon might be a result of the quantitative dynamics of AaV transcription or this virus exploiting more host machinery than the other “giants.” It is also possible that overwhelming majority of the reads originated from both infected and uninfected cells during the first lytic cycle and we see proportionally less viral reads within the sequence space. In the future a better understanding of virus–host interactions, the infectivity of AaV particles, and the availability of infection transcriptomes from other giant viruses will help resolve this question.

The enrichment of the octamer motif “AAAAATGA” in the genes expressed within 6 h of infection suggests that this is the early promoter motif of AaV (Supplementary Figure 4A). This motif is similar to the reported Mimivirus and *C. roenbergensis* virus early promoter motif “AAAATTGA” (Fischer et al., 2010; Legendre et al., 2010), with only one mismatch (at the fifth position). In contrast, AaV early promoter motif was not similar to that of algal virus PBCV-1, which is a member of *Phycodnaviridae*. This may indicate a degree of evolutionary conservation of the early promoter motif across the *Mimiviridae* clade. Analyzing the genome sequences of other *Mimiviridae* members might provide further support to this observation.

Giant viruses contain a diverse array of genes and even functional proteins (e.g., Fischer et al., 2014) within their capsids, which they use to transform their host’s cellular environment. In case of AaV, the immediately expressed genes included both proteases and ubiquitin ligases, which possibly participate in degrading the host proteins. Several transcription factors and RNA polymerase subunits were also expressed at the same time, which likely allowed transcription of the virus genes independent of at least some of the host’s apparatus (Supplementary Table 2). The expression of carbohydrate metabolism genes (putatively acquired by HGT) in AaV also leads to some intriguing possibilities. It is known that unsaturated glucuronyl hydrolases remove the terminal unsaturated sugar from the oligosaccharide products released by polysaccharide lyases (Jongkees and Withers, 2011). Healthy *Aureococcus* cells are surrounded by a fibrous glycocalyx, which is absent from the virus-infected cells (Gastrich et al., 1998): it is thus compelling to speculate that the role of a polysaccharide lyase and glucuronyl hydrolase during infection is to make the cell membrane accessible for virus attachment. Analysis of the proteome of AaV particles will be necessary to determine whether these proteins are packaged within the capsid which could be used to target the host membrane.

### The *Aureococcus* “Virocell” – Transcriptional Remodeling Upon Virus Infection

One observation within this study was a rapid transcriptional response of the host after virus treatment (**Figure 3**). In part the differentially expressed gene pool might reflect host defense response to virus attack. However, this response also potentially includes genes that are rapidly manipulated by the virus to transform the cellular environment in favor of virus propagation. This rapid change at transcription level perhaps represents how the transformation of a healthy cell into a “virocell” (Forterre, 2011) is initiated. An interesting observation was the dramatic reduction in number of deregulated genes at 1-h time point. Our analyses indicate that this phenomenon is not a result of human error or sample mislabeling. This likely has a biological basis, since control and infected RNA-seq data at 1-h show higher similarity in nMDS space compared to other control and infection sample pairs (Supplementary Figure 5). Underlying cause of this observation is yet to be determined. We observed deregulation of numerous genes and cellular processes across all time points but 1-h, which provide important insights into the mechanistic basis of AaV propagation. Higher expression of ribosome, translation, and endoplasmic reticulum-related processes possibly resulted in elevation of protein synthesis, whereas suppression of Golgi vesicle-associated processes indicate that AaV infection affected sorting of cellular proteins to their respective destinations (Rodriguez-Boulan and Müsch, 2005). Increased energy requirement for viral replication was indicated by overrepresentation of mitochondria and cellular respiration-related GO terms (Sanchez and Lagunoff, 2015). All the *Aureococcus* genes annotated within the GO category “unsaturated fatty acid metabolic processes” are delta fatty acid desaturases – enzymes incorporating desaturation (carbon/carbon double bond) in fatty acids. These enzymes have important role in maintaining membrane fluidity during stress (Aguilar and de Mendoza, 2006). Some cyanophages encode for fatty acid desaturases (Roitman et al., 2017), and it has been suggested that maintenance of membrane fluidity is critical for assembly and activity of membrane proteins like D1 protein in photosystem II (Gombos et al., 1997). One of the possible effect of deregulation of these genes upon AaV infection could be compromised structure and function of the photosynthetic apparatus in the infected *Aureococcus* cells. The deregulation of the GBPs and phagosome pathway in virus infected cells is interesting since GBPs are known to transport antimicrobial peptides, NADPH oxidase components, and the machinery of autophagy in the phagosomal compartments (Dupont and Hunter, 2012). These observations indicate that GBPs might be part of a defense network in *Aureococcus* against infection. Circumvention of this network is probably crucial in establishing successful infection by AaV.

The possible roles of polyamines in propagation of several viruses (Raina et al., 1981; Baumann et al., 2007) have been previously noted – including their role in neutralizing the negatively charged nucleic acid inside the capsid (Gibson and Roizman, 1971). Interestingly, *Chlorella* virus PBCV-1, NY-2A, and MT325 encode a complete polyamine biosynthetic pathway in their genomes, enabling them to synthesize homospermidine and putrescine (Baumann et al., 2007). While no polyamine biosynthesis genes could be located on AaV genome, increased polyamine biosynthesis in infected *Aureococcus* cells (Supplementary Figure 8) alongside adoption of polyamine synthesis genes by *Chlorella* viruses might indicate a widespread role of polyamines in giant virus life cycles.

Relative to competing plankton, *Aureococcus* has a larger number of nuclear-encoded LHC proteins, which augment the photosynthetic reaction center in collecting light energy (Gobler et al., 2011). It is known that lower light level can delay the virus-mediated lysis of *Aureococcus*, and photosynthetic efficiency is not significantly different between infected and non-infected cultures within 24-h of infection (Gobler et al., 2007). However, the molecular basis of how AaV infection can influence the host photosynthetic capacity is largely unknown. Immediately after infection, transcripts for photosynthesis-related processes proportionally decreased relative to control, with increasing number of LHC protein-encoding genes being down-regulated as infection progressed (Supplementary Figure 9). Along with the LHC genes, isoprenoid biosynthesis genes were also downregulated. Isoprenoids are an important functional and structural part of the photosynthetic apparatus and photosynthetic electron carriers, with roles in regulating the fluidity of photosynthetic membranes. (Havaux, 1998). Also, isoprenoid compounds like zeaxanthin and β-carotene are involved in photoprotection – they dissipate excessive light energy through heat (Peñuelas and Munné-Bosch, 2005). A possible consequence of the suppression of isoprenoid biosynthesis genes could be structural damage of the photosynthetic components of the infected cells. AaV propagation was found to be adversely affected at low light – with cultures incubated in low light (∼3 μmol quanta m^-2^ s^-1^) taking more than 7 days to be reduced to <10^4^ cells/ml compared to high light (∼110 μmol quanta m^-2^ s^-1^) incubated culture, which took 3 days to be reduced to similar concentration (Gobler et al., 2007). Down-regulation of photosynthesis was also observed in *Chlorella* upon infection with PBCV-1 (Seaton et al., 1995) and *Heterosigma akashiwo* infected by either RNA or DNA viruses (Philippe et al., 2003). Photosynthesis was found to be down-regulated in a wide range of plants in response to pathogen invasion (e.g., virus and bacteria) (Bilgin et al., 2010) and was suggested to be an adaptive response to biotic attack. It is important to note that down-regulation of gene expression doesn’t necessarily mean immediate loss of function – specifically, the proteins involved in light reaction might have a long functional lifetime (Bilgin et al., 2010). Thus, the actual effect of immediate down-regulation of photosynthesis gene expression on viral attack remains to be elucidated. It was proposed that slow turnover of many photosynthesis-related proteins allows the host to redirect resources for immediate defense mechanisms without dramatically reducing its photosynthetic capacity (Bilgin et al., 2010). High light requirement of the virus and the capacity of *Aureococcus* to grow in a low light environment might itself act as a natural defense mechanism at the community level, where delayed virus production can eventually lead to fewer host–virus contacts and infection.

In photosynthetic organisms, different chlorophyll precursors are formed as part of its biosynthetic pathway. However, accumulation of such precursors, especially protoporphyrin IX, can lead to photosensitivity (Inagaki et al., 2015). In the presence of light, the chlorophyll precursors with a porphyrin structure interact with triplet oxygen molecule to produce highly reactive singlet oxygen, which in turn can catalyze peroxidation of lipids and other cellular macromolecules (Rüdiger, 2009). It has also been demonstrated that various porphyrin derivatives might have broad antiviral activity – however, the activity is mostly extracellular. For example, an alkylated porphyrin (chlorophyllide) was found to cause damage to the hepatitis B-virus capsid (Guo et al., 2011), leading to loss of virion DNA. Thus, increase in porphyrin synthesis-related gene expression might lead to increased porphyrin concentrations in AaV-infected cells, elevating the oxidative stress and making the cellular environment hostile for the invading viral DNA (Mock et al., 1998). Porphyrin compounds might also damage the components of the infected cells that are important for completion of AaV replication cycle. Intriguingly, AaV encodes a pheophorbide A oxygenase gene (AaV_372) which is a key regulator in heme/chlorophyll breakdown (Hörtensteiner, 2013). Transcripts for this gene were initially detected at 6-h post-infection (Supplementary Table 1). Presence of this transcript poses the interesting possibility that AaV might use it to counteract the oxidative damage induced by high porphyrin biosynthesis. Together, up-regulation of porphyrin biosynthesis genes and concomitant down-regulation of DNA photolyase (**Figure 4**) might work as one of the first lines of host defense – an oxidative intracellular environment with a suppression of DNA repair activity. Recently, photolyase was reported to be part of the CroV proteome (Fischer et al., 2014). It was also found that majority of the packaged proteins, including photolyase, were late proteins (Fischer et al., 2014). A photolyase is also encoded into AaV genome, which was found to be expressed late (12 h post-infection). It is possible that proteins involved in subversion of host defense are also packaged in the AaV virion. AaV also encodes MutS (Moniruzzaman et al., 2014), a protein putatively involved in DNA MMR (Wilson et al., 2014). Diverse viruses are known to exploit the cellular DNA damage repair machineries (e.g., Weinbauer et al., 1997). In herpes simplex virus-1 (HSV-1), cellular MMR proteins are crucial for efficient replication which is evidenced by accumulation of these proteins in the replication centers (Ogata et al., 2011). The role of host MMR system in giant virus replication is unknown; however, MutS homologs are present in all the known members of the *Mimiviridae* family (Ogata et al., 2011; Wilson et al., 2014). The modulation of host MMR gene expression by AaV (Supplementary Figure 7) indicates that both virus- and host-encoded MMR machineries are important for successful propagation of AaV and likely other *Mimiviridae* members.

The majority of the selenoproteins characterized to date have redox-active functions; however, they can also have a wide range of biological roles (Labunskyy et al., 2014). Some viruses can encode Sec-containing proteins, with bioinformatics evidence provided for several mammalian viruses (Taylor et al., 1997): indeed, a Sec-containing GPx experimentally characterized in HIV-1 (Zhao et al., 2000). Given the unrivaled compendium of *Aureococcus* selenoprotein encoding genes within the eukaryotic domain, we were interested in how the expressions of these genes would be modulated by AaV infection and their possible role in virus propagation. Our study indicates that a number of up-regulated selenoprotein genes are possibly involved in viral protein synthesis and preventing oxidation of these viral proteins, especially during the late phase of infection (**Figure 6**). It has been demonstrated that Sec-containing MSR are efficient, showing 10–50-fold higher enzymatic activity compared to the Cys-containing MSRs (Kim et al., 2006). Additionally, selenoproteins deemed crucial in regulating the redox state of the cells (e.g., GPx and dehydroascorbate reductase) were also overexpressed during infection. The cellular pro-/antioxidant balance is a highly complex process, involving a cascade of enzymatic activity and interconnected pathways. As was aptly put by Schwarz (1996), “it is difficult to distinguish between association and causation as well as between primary and secondary effects of a given virus on ROS mediated cellular injury.” In EhV, observations from transcriptome data coupled to targeted experiments revealed an elevated production of GSH along with H_2_O_2_ accumulation in the infected cells. Interestingly, addition of a peroxidase inhibitor (esculetin) or a H_2_O_2_ inhibitor (potassium iodide) to the infected cells dramatically reduced cell death and virus production, indicating a complex yet important role of virus-mediated ROS and antioxidant modulation of host cells for successful virus production (Sheyn et al., 2016). While in AaV-infected cultures we observed up-regulation of a superoxide dismutase gene, suggesting accumulation of H_2_O_2_ (no catalase is annotated in *Aureococcus* genome), no further inference can be made on its role without targeted experiments. However, up-regulation of a large number of selenoprotein genes involved in protein damage repair, folding, and other redox functions can indeed be interpreted as a response to increase in cellular oxidative stress under which viral protein synthesis and assembly were likely progressing. Additionally, increase in Sec biosynthesis and its conversion to other amino acids (Supplementary Figure 10) point to the possibility of increased requirement of selenium for the infected cells. Although we observed overexpression of phosphate uptake genes in the infected cells, it is not possible to link this with increased selenium uptake without further experiments. Indeed, this could simply imply that AaV proliferation may require elevated concentration of phosphate in cell. Phosphate depletion has been shown to massively reduce the burst size of PpV in *Phaeocystis pouchetii* (Carreira et al., 2013), PBCV in *Chlorella* sp. (Carreira et al., 2013), and MpV in *Micromonas pusilla* (Maat et al., 2014). Further studies will be necessary to elucidate the effect of selenium deficiency and mechanism of selenium uptake during the replication of AaV and other algal viruses.

## Conclusion

Host–virus interactions at nanoscale eventually shape ecosystem processes at geographical scales (Brussaard et al., 2008). Resolving the molecular aspects of ecologically relevant host–virus interactions is critical to understand the role of viruses in the biogeochemical processes, as well as the factors that drive the co-evolution of virus–host systems (Rosenwasser et al., 2016). In this study, we gained insights into the transcriptomic response of a harmful alga upon infection by a giant virus. The ultimate fate of a cell going through lytic infection is to produce progeny viruses, which is accomplished through a different transcriptomic and metabolic trajectory relative to a healthy cell. The most likely outcome of this massive transcriptional response is that a reprogrammed metabolic profile-specific metabolites might regulate viral replication and might be incorporated in the virion particles. The altered metabolism of virus-infected cells might even influence large-scale ecological processes; for example, differential uptake or release of specific compounds might alter the nutrient dynamics, thereby affecting the coexisting microbial communities (Ankrah et al., 2014). This study will provide an important foundation to generate and test new hypotheses regarding individual metabolic or regulatory processes that can have important biogeochemical consequences, and perhaps more importantly, place the “virocell” into a better ecological context.

## Author Contributions

MM and SW designed the experiments and secured funding. MM, EG, and SW carried out the experiments and analysis and wrote the paper.

## Conflict of Interest Statement

The authors declare that the research was conducted in the absence of any commercial or financial relationships that could be construed as a potential conflict of interest.

## Footnotes

1 http://genome.jgi-psf.org/Auran1/Auran1.home.html

## References

[B1] Aguilar P. S. de Mendoza D. (2006). Control of fatty acid desaturation: a mechanism conserved from bacteria to humans. *Mol. Microbiol.* 62 1507–1514. 10.1111/j.1365-2958.2006.05484.x 17087771

[B2] Alikhan N.-F. Petty N. Ben Zakour N. Beatson S. (2011). BLAST ring image generator (BRIG): simple prokaryote genome comparisons. *BMC Genomics* 12:402. 10.1186/1471-2164-12-402 21824423 PMC3163573

[B3] Angly F. Rodriguez-Brito B. Bangor D. Mcnairnie P. Breitbart M. Salamon P. (2005). PHACCS, an online tool for estimating the structure and diversity of uncultured viral communities using metagenomic information. *BMC Bioinformatics* 6:41. 10.1186/1471-2105-6-41 15743531 PMC555943

[B4] Ankrah N. Y. May A. L. Middleton J. L. Jones D. R. Hadden M. K. Gooding J. R. (2014). Phage infection of an environmentally relevant marine bacterium alters host metabolism and lysate composition. *ISME J.* 8 1089–1100. 10.1038/ismej.2013.216 24304672 PMC3996693

[B5] Bailey T. L. Boden M. Buske F. A. Frith M. Grant C. E. Clementi L. (2009). MEME Suite: tools for motif discovery and searching. *Nucleic Acids. Res.* 37 W202–W208. 10.1093/nar/gkp335 19458158 PMC2703892

[B6] Bailey T. L. Elkan C. (1994). Fitting a mixture model by expectation maximization to discover motifs in biopolymers. *Proc. Int. Conf. Intell. Syst. Mol. Biol.* 2 28–36. 7584402

[B7] Baumann S. Sander A. Gurnon J. R. Yanai-Balser G. M. Van Etten J. L. Piotrowski M. (2007). Chlorella viruses contain genes encoding a complete polyamine biosynthetic pathway. *Virology* 360 209–217. 10.1016/j.virol.2006.10.010 17101165 PMC1971760

[B8] Benjamini Y. Hochberg Y. (1995). Controlling the false discovery rate: a practical and powerful approach to multiple testing. *J. R. Stat. Soc. B (Methodological)* 57 289–300.

[B9] Bilgin D. D. Zavala J. A. Zhu J. I. N. Clough S. J. Ort D. R. Delucia E. H. (2010). Biotic stress globally downregulates photosynthesis genes. *Plant Cell Environ.* 33 1597–1613. 10.1111/j.1365-3040.2010.02167.x 20444224

[B10] Blanc G. Mozar M. Agarkova I. V. Gurnon J. R. Yanai-Balser G. Rowe J. M. (2014). Deep RNA sequencing reveals hidden features and dynamics of early gene transcription in *Paramecium bursaria* Chlorella Virus 1. *PLoS One* 9:e90989. 10.1371/journal.pone.0090989 24608750 PMC3946568

[B11] Brown C. M. Bidle K. D. (2014). Attenuation of virus production at high multiplicities of infection in *Aureococcus anophagefferens*. *Virology* 466-467 71–81. 10.1016/j.virol.2014.07.023 25104555

[B12] Brussaard C. P. Wilhelm S. W. Thingstad F. Weinbauer M. G. Bratbak G. Heldal M. (2008). Global-scale processes with a nanoscale drive: the role of marine viruses. *ISME J.* 2 575–578. 10.1038/ismej.2008.31 18385772

[B13] Carreira C. Heldal M. Bratbak G. (2013). Effect of increased pCO2 on phytoplankton–virus interactions. *Biogeochemistry* 114 391–397. 10.1007/s10533-011-9692-x

[B14] Chang J. H. Tong L. (2012). Mitochondrial poly(A) polymerase and polyadenylation. *Biochim. Biophys. Acta* 1819 992–997. 10.1016/j.bbagrm.2011.10.012 22172994 PMC3307840

[B15] Clarke K. R. (1993). Non-parametric multivariate analyses of changes in community structure. *Aust. J. Ecol.* 18 117–143. 10.1111/j.1442-9993.1993.tb00438.x

[B16] Claverie J. M. Abergel C. (2010). Mimivirus: the emerging paradox of quasi-autonomous viruses. *Trends Genet.* 26 431–437. 10.1016/j.tig.2010.07.003 20696492

[B17] Danielson U. H. Esterbauer H. Mannervik B. (1987). Structure-activity relationships of 4-hydroxyalkenals in the conjugation catalysed by mammalian glutathione transferases. *Biochem. J.* 247: 707. 10.1042/bj2470707 3426557 PMC1148470

[B18] Dupont C. D. Hunter C. A. (2012). Guanylate-binding proteins: niche recruiters for antimicrobial effectors. *Immunity* 37 191–193. 10.1016/j.immuni.2012.08.005 22921115

[B19] Filee J. Siguier P. Chandler M. (2007). I am what I eat and I eat what I am: acquisition of bacterial genes by giant viruses. *Trends Genet.* 23 10–15. 10.1016/j.tig.2006.11.002 17109990

[B20] Fischer M. G. Allen M. J. Wilson W. H. Suttle C. A. (2010). Giant virus with a remarkable complement of genes infects marine zooplankton. *Proc. Natl. Acad. Sci. U.S.A.* 107 19508–19513. 10.1073/pnas.1007615107 20974979 PMC2984142

[B21] Fischer M. G. Kelly I. Foster L. J. Suttle C. A. (2014). The virion of *Cafeteria roenbergensis* Virus (CroV) contains a complex suite of proteins for transcription and DNA repair. *Virology* 466–467, 82–94. 10.1016/j.virol.2014.05.029 24973308

[B22] Forterre P. (2011). Manipulation of cellular syntheses and the nature of viruses: the virocell concept. *C. R. Chim.* 14 392–399. 10.1016/j.crci.2010.06.007

[B23] Gastrich M. Leigh-Bell J. Gobler C. Roger Anderson O. Wilhelm S. W. Bryan M. (2004). Viruses as potential regulators of regional brown tide blooms caused by the alga *Aureococcus anophagefferens. Estuaries* 27 112–119. 10.1007/BF02803565

[B24] Gastrich M. D. Anderson O. R. Benmayor S. S. Cosper E. M. (1998). Ultrastructural analysis of viral infection in the brown-tide alga, *Aureococcus anophagefferens* (Pelagophyceae). *Phycologia* 37 300–306. 10.2216/i0031-8884-37-4-300.1

[B25] Gibson W. Roizman B. (1971). Compartmentalization of spermine and spermidine in the Herpes Simplex virion. *Proc. Natl. Acad. Sci. U.S.A.* 68 2818–2821. 10.1073/pnas.68.11.2818 5288261 PMC389533

[B26] Gobler C. J. Anderson O. R. Gastrich M. D. Wilhelm S. W. (2007). Ecological aspects of viral infection and lysis in the harmful brown tide alga *Aureococcus anophagefferens*. *Aquat. Microb. Ecol.* 47 25–36. 10.3354/ame047025

[B27] Gobler C. J. Berry D. L. Dyhrman S. T. Wilhelm S. W. Salamov A. Lobanov A. V. (2011). Niche of harmful alga *Aureococcus anophagefferens* revealed through ecogenomics. *Proc. Natl. Acad. Sci. U.S.A.* 108 4352–4357. 10.1073/pnas.1016106108 21368207 PMC3060233

[B28] Gobler C. J. Lonsdale D. J. Boyer G. L. (2005). A review of the causes, effects, and potential management of harmful brown tide blooms caused by *Aureococcus anophagefferens* (Hargraves et Sieburth). *Estuaries* 28 726–749. 10.1007/BF02732911

[B29] Gombos Z. Kanervo E. Tsvetkova N. Sakamoto T. Aro E. M. Murata N. (1997). Genetic enhancement of the ability to tolerate photoinhibition by introduction of unsaturated bonds into membrane glycerolipids. *Plant Physiol.* 115 551–559. 10.1104/pp.115.2.551 12223823 PMC158514

[B30] Guo H. Pan X. Mao R. Zhang X. Wang L. Lu X. (2011). Alkylated porphyrins have broad antiviral activity against Hepadnaviruses, Flaviviruses, Filoviruses, and Arenaviruses. *Antimicrob. Agents Chemother.* 55 478–486. 10.1128/AAC.00989-10 21135183 PMC3028764

[B31] Hallegraeff G. M. Anderson D. M. Cembella A. D. (eds) (2003). *Manual on Harmful Marine Microalgae* Vol. 11 Paris: UNESCO, p. 792.

[B32] Havaux M. (1998). Carotenoids as membrane stabilizers in chloroplasts. *Trends Plant Sci.* 3 147–151. 10.1016/S1360-1385(98)01200-X

[B33] Hörtensteiner S. (2013). Update on the biochemistry of chlorophyll breakdown. *Plant Mol. Biol.* 82 505–517. 10.1007/s11103-012-9940-z 22790503

[B34] Inagaki N. Kinoshita K. Kagawa T. Tanaka A. Ueno O. Shimada H. (2015). Phytochrome B mediates the regulation of chlorophyll biosynthesis through transcriptional regulation of *ChlH* and *GUN4* in rice seedlings. *PLoS One* 10:e0135408. 10.1371/journal.pone.0135408 26270815 PMC4536196

[B35] Iyer L. M. Aravind L. Koonin E. V. (2001). Common origin of four diverse families of large eukaryotic DNA viruses. *J. Virol.* 75 11720–11734. 10.1128/JVI.75.23.11720-11734.2001 11689653 PMC114758

[B36] Jongkees S. A. Withers S. G. (2011). Glycoside cleavage by a new mechanism in unsaturated glucuronyl hydrolases. *J. Am. Chem. Soc.* 133 19334–19337. 10.1021/ja209067v 22047074

[B37] Keller M. D. Bellows W. K. Guillard R. R. L. (1988). Microwave treatment for sterilization of phytoplankton culture media. *J. Exp. Mar. Biol. Ecol.* 117 279–283. 10.1016/0022-0981(88)90063-9

[B38] Kim H.-Y. Fomenko D. E. Yoon Y.-E. Gladyshev V. N. (2006). Catalytic advantages provided by selenocysteine in methionine-S-sulfoxide reductases. *Biochemistry* 45 13697–13704. 10.1021/bi0611614 17105189 PMC2519125

[B39] Koonin E. V. Yutin N. (2010). Origin and evolution of eukaryotic large nucleo-cytoplasmic DNA viruses. *Intervirology* 53 284–292. 10.1159/000312913 20551680 PMC2895762

[B40] Labunskyy V. M. Hatfield D. L. Gladyshev V. N. (2014). Selenoproteins: molecular pathways and physiological roles. *Physiol. Rev.* 94 739–777. 10.1152/physrev.00039.2013 24987004 PMC4101630

[B41] Labunskyy V. M. Yoo M. H. Hatfield D. L. Gladyshev V. N. (2009). Sep15, a thioredoxin-like selenoprotein, is involved in the unfolded protein response and differentially regulated by adaptive and acute ER stresses. *Biochemistry* 48 8458–8465. 10.1021/bi900717p 19650649 PMC2778599

[B42] Lang A. S. Rise M. L. Culley A. I. Steward G. F. (2009). RNA viruses in the sea. *FEMS Microbiol. Rev.* 33 295–323. 10.1111/j.1574-6976.2008.00132.x 19243445

[B43] Lazard M. Blanquet S. Fisicaro P. Labarraque G. Plateau P. (2010). Uptake of selenite by *Saccharomyces cerevisiae* involves the high and low affinity orthophosphate transporters. *J. Biol. Chem.* 285 32029–32037. 10.1074/jbc.M110.139865 20688911 PMC2952204

[B44] Legendre M. Audic S. Poirot O. Hingamp P. Seltzer V. Byrne D. (2010). mRNA deep sequencing reveals 75 new genes and a complex transcriptional landscape in Mimivirus. *Genome Res.* 20 664–674. 10.1101/gr.102582.109 20360389 PMC2860168

[B45] Liang T. J. (2009). Hepatitis B: the virus and disease. *Hepatology* 49 S13–S21. 10.1002/hep.22881 19399811 PMC2809016

[B46] Luo W. Friedman M. S. Shedden K. Hankenson K. D. Woolf P. J. (2009). GAGE: generally applicable gene set enrichment for pathway analysis. *BMC Bioinformatics* 10:161. 10.1186/1471-2105-10-161 19473525 PMC2696452

[B47] Maat D. S. Crawfurd K. J. Timmermans K. R. Brussaard C. P. D. (2014). Elevated CO2 and phosphate limitation favor *Micromonas pusilla* through stimulated growth and reduced viral impact. *Appl. Environ. Microbiol.* 80 3119–3127. 10.1128/AEM.03639-13 24610859 PMC4018922

[B48] Maere S. Heymans K. Kuiper M. (2005). BiNGO: a Cytoscape plugin to assess overrepresentation of Gene Ontology categories in biological networks. *Bioinformatics* 21 3448–3449. 10.1093/bioinformatics/bti551 15972284

[B49] Mock H.-P. Keetman U. Kruse E. Rank B. Grimm B. (1998). Defense responses to tetrapyrrole-induced oxidative stress in transgenic plants with reduced uroporphyrinogen decarboxylase or coproporphyrinogen oxidase activity. *Plant Physiol.* 116 107–116. 10.1104/pp.116.1.107

[B50] Moniruzzaman M. Lecleir G. R. Brown C. M. Gobler C. J. Bidle K. D. Wilson W. H. (2014). Genome of brown tide virus (AaV), the little giant of the Megaviridae, elucidates NCLDV genome expansion and host–virus coevolution. *Virology* 466-467 60–70. 10.1016/j.virol.2014.06.031 25035289

[B51] Moniruzzaman M. Wurch L. L. Alexander H. Dyhrman S. T. Gobler C. J. Wilhelm S. W. (2017). Virus-host relationships of marine single-celled eukaryotes resolved from metatranscriptomics. *Nat. Commun.* 8:16054. 10.1038/ncomms16054 28656958 PMC5493757

[B52] Monnier A. Liverani S. Bouvet R. Jesson B. Smith J. Q. Mosser J. (2010). Orchestrated transcription of biological processes in the marine picoeukaryote Ostreococcus exposed to light/dark cycles. *BMC Genomics* 11:192. 10.1186/1471-2164-11-192 20307298 PMC2850359

[B53] Moskovitz J. (2005). Methionine sulfoxide reductases: ubiquitous enzymes involved in antioxidant defense, protein regulation, and prevention of aging-associated diseases. *Biochim. Biophys. Acta* 1703 213–219. 10.1016/j.bbapap.2004.09.003 15680229

[B54] Noble R. T. Fuhrman J. A. (1998). Use of SYBR Green I for rapid epifluorescence counts of marine viruses and bacteria. *Aquat. Microb. Ecol.* 14 113–118. 10.3354/ame014113

[B55] Noctor G. Foyer C. H. (1998). Ascorbate and glutathione: keeping active oxygen under control. *Annu. Rev. Plant Physiol. Plant Mol. Biol.* 49 249–279. 10.1146/annurev.arplant.49.1.249 15012235

[B56] Obornik M. Green B. R. (2005). Mosaic origin of the heme biosynthesis pathway in photosynthetic eukaryotes. *Mol. Biol. Evol.* 22 2343–2353. 10.1093/molbev/msi230 16093570

[B57] Ogata H. Ray J. Toyoda K. Sandaa R.-A. Nagasaki K. Bratbak G. (2011). Two new subfamilies of DNA mismatch repair proteins (MutS) specifically abundant in the marine environment. *ISME J.* 5 1143–1151. 10.1038/ismej.2010.210 21248859 PMC3146287

[B58] Ong H. C. Wilhelm S. W. Gobler C. J. Bullerjahn G. Jacobs M. A. Mckay J. (2010). Analyses of the complete chloroplast genome sequences of two members of the pelagophyceae: *Aureococcus anophagefferens* CCMP1984 and *Aureoumbra lagunensis* CCMP15071. *J. Phycol.* 46 602–615. 10.1111/j.1529-8817.2010.00841.x

[B59] Ortmann A. C. Suttle C. A. (2009). Determination of virus abundance by epifluorescence microscopy. *Methods Mol. Biol.* 501 87–95. 10.1007/978-1-60327-164-6_10 19066814

[B60] Peñuelas J. Munné-Bosch S. (2005). Isoprenoids: an evolutionary pool for photoprotection. *Trends Plant Sci.* 10 166–169. 10.1016/j.tplants.2005.02.005 15817417

[B61] Philippe J. Janice E. L. Curtis A. S. Paul J. H. (2003). Effects of viral infection on photosynthetic processes in the bloom-forming alga Heterosigma akashiwo. *Aquat. Microb. Ecol.* 31 9–17. 10.3354/ame031009

[B62] R Core Team (2013). *R: A Language and Environment for Statistical Computing*. Vienna: R Foundation for Statistical Computing.

[B63] Raina A. Tuomi K. Mantyjarvi R. (1981). Roles of polyamines in the replication of animal viruses. *Med. Biol.* 59 428–432. 6279979

[B64] Raoult D. Audic S. Robert C. Abergel C. Renesto P. Ogata H. (2004). The 1.2-Megabase genome sequence of Mimivirus. *Science* 306 1344–1350. 10.1126/science.1101485 15486256

[B65] Reinbothe S. Reinbothe C. Apel K. Lebedev N. (1996). Evolution of chlorophyll biosynthesis—the challenge to survive photooxidation. *Cell* 86 703–705. 10.1016/S0092-8674(00)80144-0 8797817

[B66] Renesto P. Abergel C. Decloquement P. Moinier D. Azza S. Ogata H. (2006). Mimivirus giant particles incorporate a large fraction of anonymous and unique gene products. *J. Virol.* 80 11678–11685. 10.1128/JVI.00940-06 16971431 PMC1642625

[B67] Robinson M. D. Mccarthy D. J. Smyth G. K. (2010). edgeR: a Bioconductor package for differential expression analysis of digital gene expression data. *Bioinformatics* 26 139–140. 10.1093/bioinformatics/btp616 19910308 PMC2796818

[B68] Rodriguez-Boulan E. Müsch A. (2005). Protein sorting in the golgi complex: shifting paradigms. *Biochim. Biophys. Acta* 1744 455–464. 10.1016/j.bbamcr.2005.04.007 15927284

[B69] Roitman S. Hornung E. Flores-Uribe J. Sharon I. Feussner I. Béjà O. (2017). Cyanophage-encoded lipid desaturases: oceanic distribution, diversity and function. *ISME J.* 12 343–355. 10.1038/ismej.2017.159 29028002 PMC5776448

[B70] Rosenwasser S. Mausz M. A. Schatz D. Sheyn U. Malitsky S. Aharoni A. (2014). Rewiring host lipid metabolism by large viruses determines the fate of *Emiliania huxley*, a bloom-forming alga in the ocean. *Plant Cell* 26 2689–2707. 10.1105/tpc.114.125641 24920329 PMC4114960

[B71] Rosenwasser S. Ziv C. Creveld S. G. Vardi A. (2016). Virocell metabolism: metabolic innovations during host-virus interactions in the ccean. *Trends Microbiol.* 24 821–832. 10.1016/j.tim.2016.06.006 27395772

[B72] Rowe J. M. Dunlap J. R. Gobler C. J. Anderson O. R. Gastrich M. D. Wilhelm S. W. (2008). Isolation of a non-phage-like lytic virus infecting *Aureococcus anophagefferens*. *J. Phycol.* 44 71–76. 10.1111/j.1529-8817.2007.00453.x 27041042

[B73] Rüdiger W. (2009). “Regulation of the late steps of chlorophyll biosynthesis,” in *Tetrapyrroles: Birth, Life and Death* eds Warren M. Smith A. (New York, NY: Springer) 263–273.

[B74] Sanchez E. L. Lagunoff M. (2015). Viral activation of cellular metabolism. *Virology* 479 609–618. 10.1016/j.virol.2015.02.038 25812764 PMC4424078

[B75] Santini S. Jeudy S. Bartoli J. Poirot O. Lescot M. Abergel C. (2013). Genome of *Phaeocystis globosa* virus PgV-16T highlights the common ancestry of the largest known DNA viruses infecting eukaryotes. *Proc. Natl. Acad. Sci. U.S.A.* 110 10800–10805. 10.1073/pnas.1303251110 23754393 PMC3696832

[B76] Schwarz K. B. (1996). Oxidative stress during viral infection: a review. *Free Radic. Biol. Med.* 21 641–649. 10.1016/0891-5849(96)00131-1 8891667

[B77] Seaton G. Lee K. Rohozinski J. (1995). Photosynthetic shutdown in Chlorella NC64A associated with the infection cycle of *Paramecium bursaria* Chlorella Virus-1. *Plant Physiol.* 108 1431–1438. 10.1104/pp.108.4.1431 12228553 PMC157521

[B78] Sheyn U. Rosenwasser S. Ben-Dor S. Porat Z. Vardi A. (2016). Modulation of host ROS metabolism is essential for viral infection of a bloom-forming Coccolithophore in the ocean. *ISME J.* 10 1742–1754. 10.1038/ismej.2015.228 26784355 PMC4918435

[B79] Short S. M. (2012). The ecology of viruses that infect eukaryotic algae. *Environ. Microbiol.* 14 2253–2271. 10.1111/j.1462-2920.2012.02706.x 22360532

[B80] Suttle C. A. (2007). Marine viruses - major players in the global ecosystem. *Nat. Rev. Microbiol.* 5 801–812. 10.1038/nrmicro1750 17853907

[B81] Tan K.-L. Board P. G. (1996). Purification and characterization of a recombinant human Theta-class glutathione transferase (GSTT2-2). *Biochem. J.* 315 727–732. 10.1042/bj3150727 8645150 PMC1217267

[B82] Tatusov R. Galperin M. Natale D. Koonin E. (2000). The COG database: a tool for genome-scale analysis of protein functions and evolution. *Nucleic Acids Res.* 28 33–36. 10.1093/nar/28.1.33 10592175 PMC102395

[B83] Taylor E. W. Nadimpalli R. G. Ramanathan C. S. (1997). Genomic structures of viral agents in relation to the biosynthesis of selenoproteins. *Biol. Trace Elem. Res.* 56 63–91. 10.1007/BF02778984 9152512

[B84] Thiagarajan V. Byrdin M. Eker A. P. Müller P. Brettel K. (2011). Kinetics of cyclobutane thymine dimer splitting by DNA photolyase directly monitored in the UV. *Proc. Natl. Acad. Sci. U.S.A.* 108 9402–9407. 10.1073/pnas.1101026108 21606324 PMC3111307

[B85] Ursini F. Maiorino M. Brigelius-Flohe R. Aumann K. D. Roveri A. Schomburg D. (1995). Diversity of glutathione peroxidases. *Methods Enzymol.* 252 38–53. 10.1016/0076-6879(95)52007-4 7476373

[B86] Vardi A. Haramaty L. Van Mooy B. A. Fredricks H. F. Kimmance S. A. Larsen A. (2012). Host-virus dynamics and subcellular controls of cell fate in a natural Coccolithophore population. *Proc. Natl. Acad. Sci. U.S.A.* 109 19327–19332. 10.1073/pnas.1208895109 23134731 PMC3511156

[B87] Vardi A. Van Mooy B. A. S. Fredricks H. F. Popendorf K. J. Ossolinski J. E. Haramaty L. (2009). Viral glycosphingolipids induce lytic infection and cell death in marine phytoplankton. *Science* 326 861–865. 10.1126/science.1177322 19892986

[B88] Veal E. A. Toone W. M. Jones N. Morgan B. A. (2002). Distinct roles for glutathione S-transferases in the oxidative stress response in *Schizosaccharomyces pombe*. *J. Biol. Chem.* 277 35523–35531. 10.1074/jbc.M111548200 12063243

[B89] Vestal D. J. Jeyaratnam J. A. (2011). The guanylate-binding proteins: emerging insights into the biochemical properties and functions of this family of large interferon-induced guanosine triphosphatase. *J. Interferon Cytokine Res.* 31 89–97. 10.1089/jir.2010.0102 21142871 PMC3021356

[B90] Weinbauer M. G. Wilhelm S. W. Suttle C. A. Garza D. R. (1997). Photoreactivation compensates for UV damage and restores infectivity to natural marine virus communities. *Appl. Environ. Microbiol.* 63 2200–2205. 9172339 10.1128/aem.63.6.2200-2205.1997 PMC168512

[B91] Wilhelm S. W. Bird J. T. Bonifer K. S. Calfee B. C. Chen T. Coy S. R. (2017). A student’s guide to giant viruses infecting small eukaryotes: from Acanthamoeba to Zooxanthellae. *Viruses* 9:E46. 10.3390/v9030046 28304329 PMC5371801

[B92] Wilhelm S. W. Coy S. R. Gann E. R. Moniruzzaman M. Stough J. M. A. (2016). Standing on the shoulders of giant viruses: five lessons learned about large viruses infecting small eukaryotes and the opportunities they create. *PLoS Pathog.* 12:e1005752. 10.1371/journal.ppat.1005752 27559742 PMC4999288

[B93] Wilhelm S. W. Suttle C. A. (1999). Viruses and nutrient cycles in the sea: viruses play critical roles in the structure and function of aquatic food webs. *Bioscience* 49 781–788. 10.2307/1313569

[B94] Wilson W. H. Gilg I. C. Duarte A. Ogata H. (2014). Development of DNA mismatch repair gene, MutS, as a diagnostic marker for detection and phylogenetic analysis of algal Megaviruses. *Virology* 466–467, 123–128. 10.1016/j.virol.2014.07.001 25063474

[B95] Wilson W. H. Schroeder D. C. Allen M. J. Holden M. T. G. Parkhill J. Barrell B. G. (2005). Complete genome sequence and lytic phase transcription profile of a Coccolithovirus. *Science* 309 1090–1092. 10.1126/science.1113109 16099989

[B96] Yutin N. Wolf Y. Raoult D. Koonin E. (2009). Eukaryotic large nucleo-cytoplasmic DNA viruses: clusters of orthologous genes and reconstruction of viral genome evolution. *Virol. J.* 6:223. 10.1186/1743-422X-6-223 20017929 PMC2806869

[B97] Zhao L. Cox A. G. Ruzicka J. A. Bhat A. A. Zhang W. Taylor E. W. (2000). Molecular modeling and in vitro activity of an HIV-1-encoded glutathione peroxidase. *Proc. Natl. Acad. Sci. U.S.A.* 97 6356–6361. 10.1073/pnas.97.12.6356 10841544 PMC18607

