# Supplementary material for: Infection by a Giant Virus (AaV) Induces Widespread Physiological Reprogramming in Aureococcus anophagefferens CCMP1984 – A Harmful Bloom Algae
Source: Front Microbiol. 2018 Apr 19;9:752. doi: 10.3389/fmicb.2018.00752 (PMC5917014; doi:10.3389/fmicb.2018.00752)
Supplement: Supplementary file 2 [file Data_Sheet_2.PDF]

The following information serves as supplemental material to be published online in conjunction with the following paper:

Infection by a giant virus (AaV) induces widespread physiological reprogramming in *Aureococcus anophagefferens* CCMP1984 – a harmful bloom algae

Mohammad Moniruzzaman[1,2], Eric R. Gann[2], Steven W. Wilhelm[2]*

1. Monterey Bay Aquarium Research Institute (MBARI), 7700 Sandholdt Road, Moss Landing, CA 95039.
2. Department of Microbiology, The University of Tennessee, Knoxville, TN USA 37996

*author for correspondence: wilhelm@utk.edu

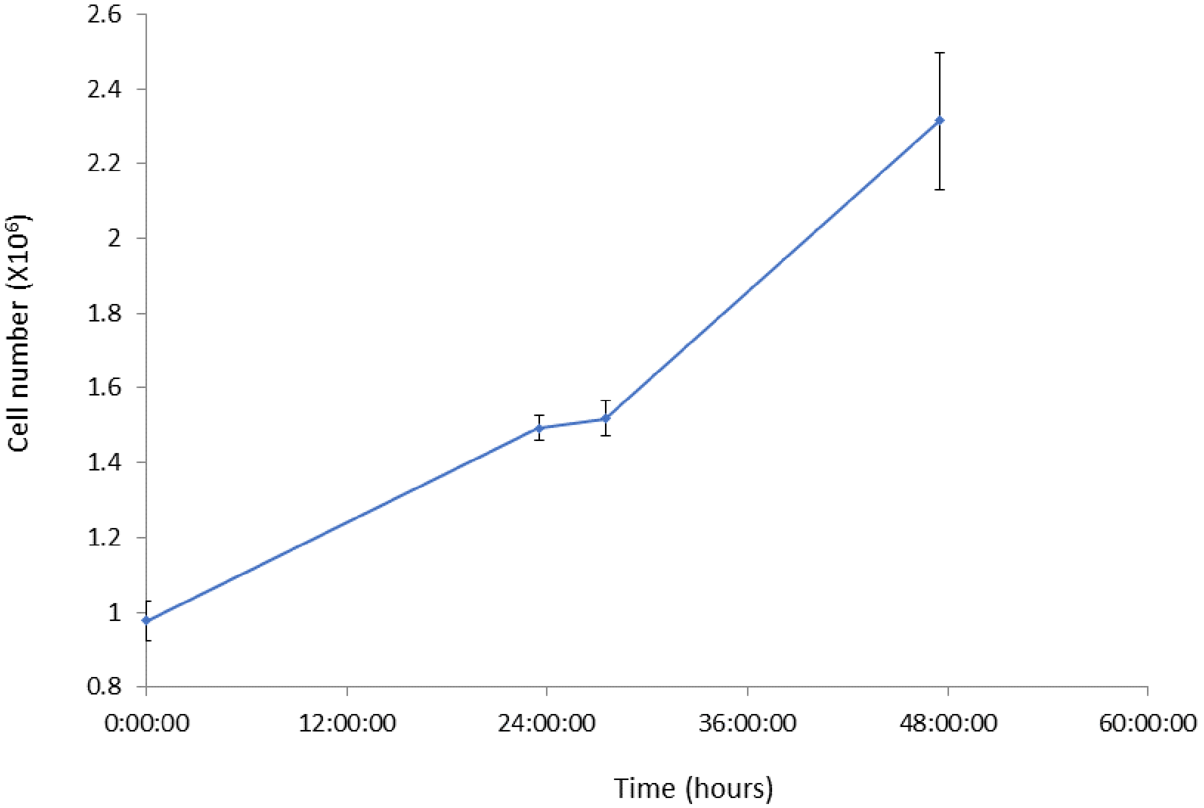

Supplementary figure 1: Growth pattern of healthy *Aureococcus anophagefferens* maintenance culture over a period of 48 hours. Data is from 3 biological replicates.

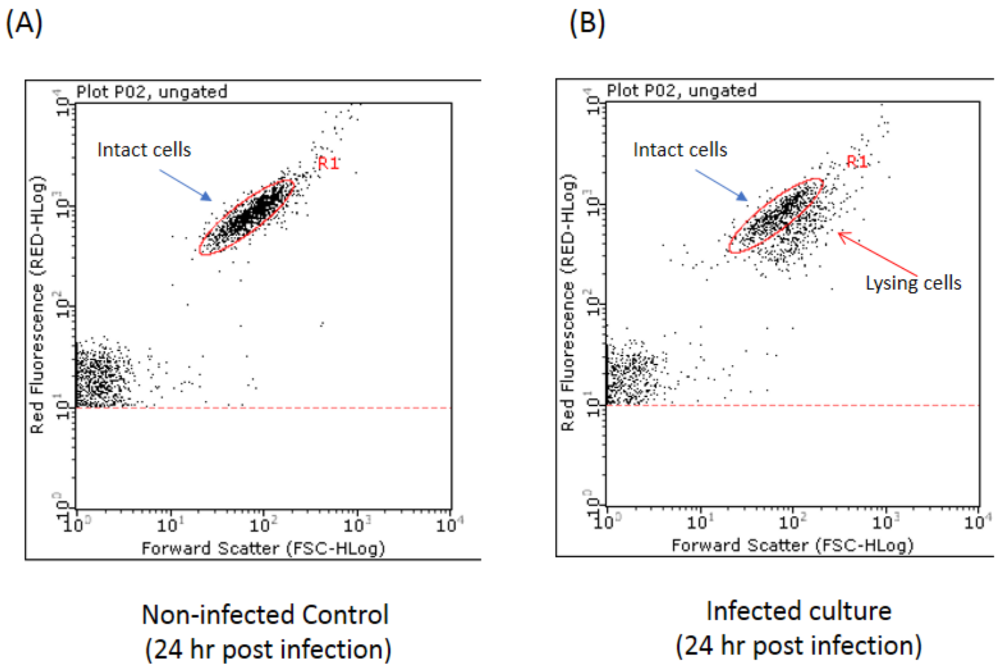

Supplementary figure 2: Flow cytometry analysis of infected and non-infected *Aureococcus* cells, showing chlorophyll fluorescence (Y-axis, 692 nm) and forward scatter signature of both intact (blue arrow) and lysing cell population (red arrow).

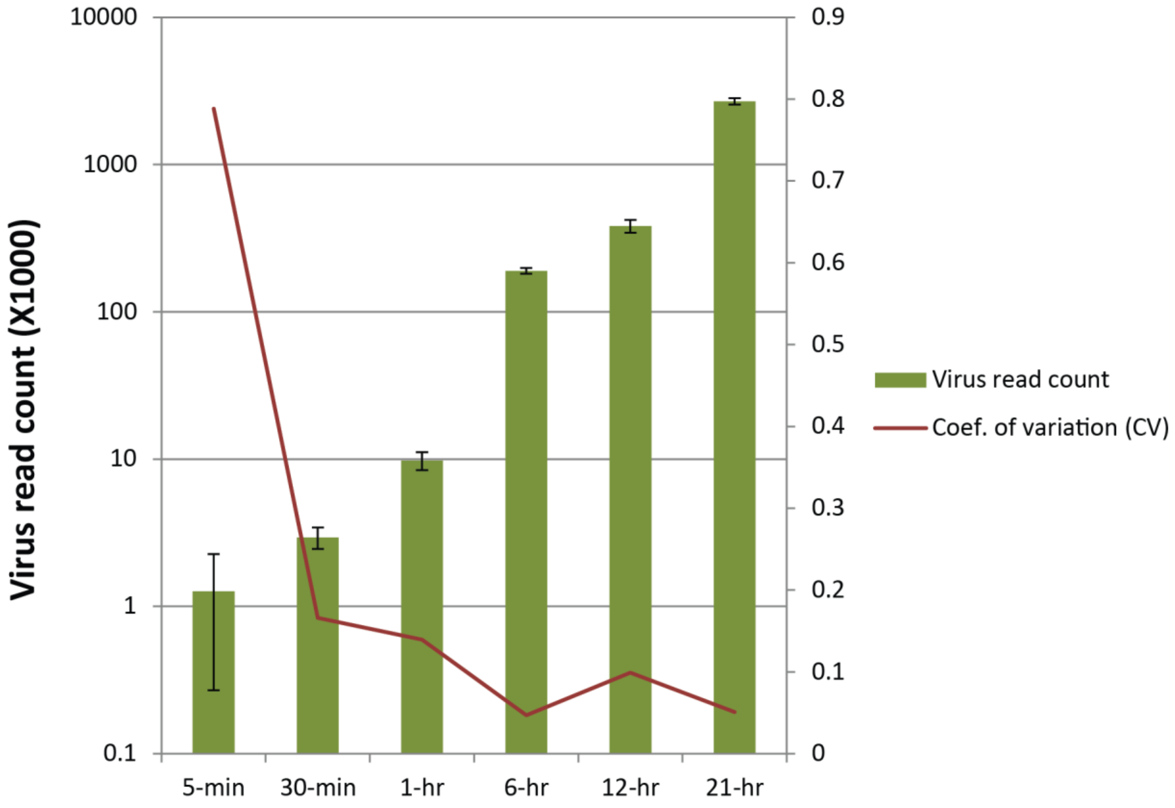

Supplementary Figure 3: Average number of reads mapped from the virus treated samples to AaV genome over time. The read counts were rarefied by library size prior averaging. The coefficient of variation for each time point is plotted on the secondary axis.

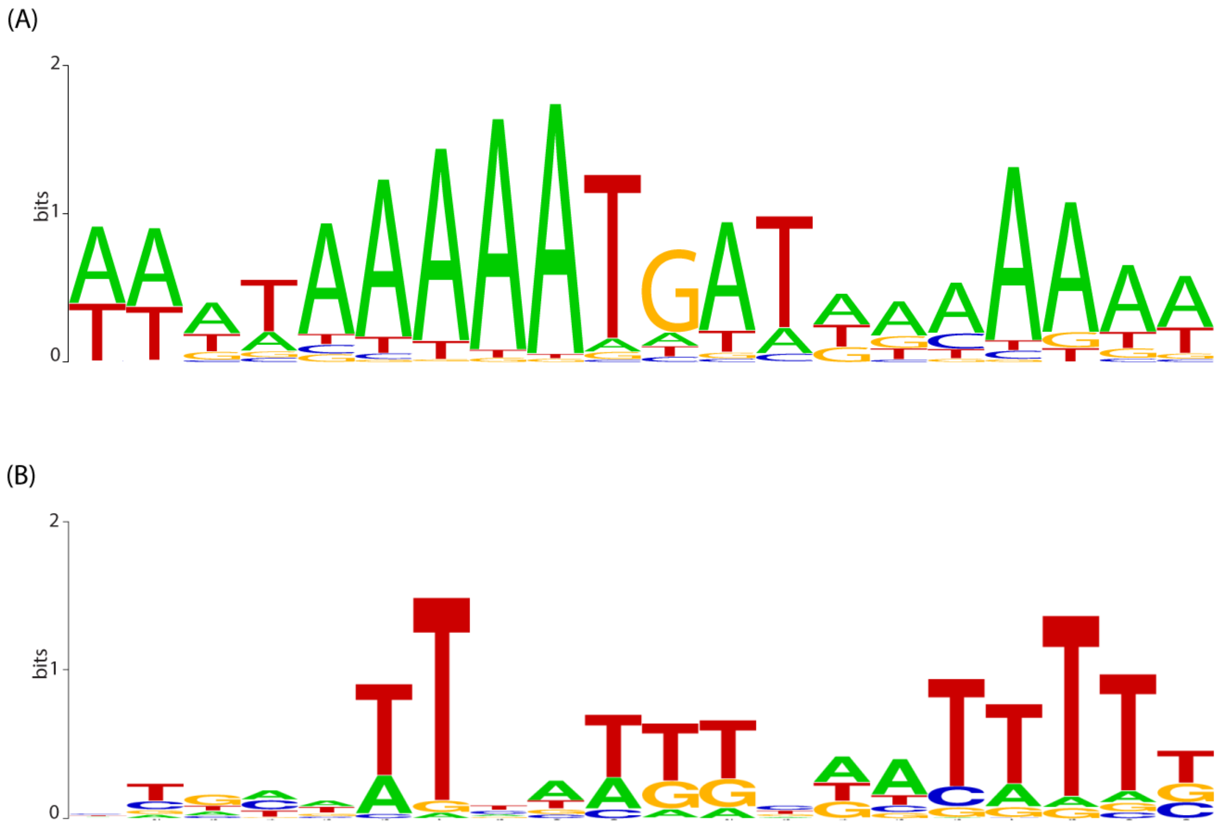

Supplementary Figure 4: Sequence logo of (A) Putative early promoter motif of AaV. (B) motif overrepresented in the ‘late’ class of genes compared to the ‘early to intermediate’ gene class in AaV.

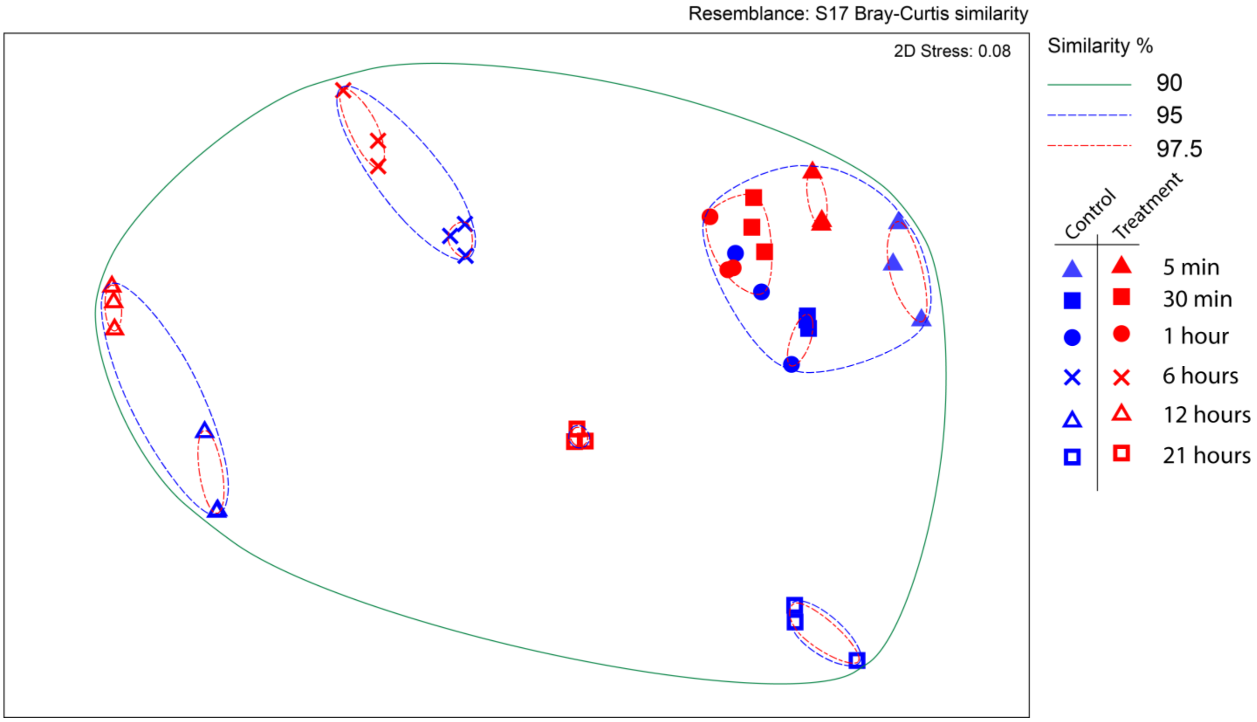

Supplementary Figure 5: nMDS plot of the TMM normalized gene expression data from all replicate samples. The circles drawn around the samples denote different level of similarity (90, 95 and 97.5%) among the samples obtained from hierarchical clustering of the samples using Bray-Curtis dissimilarity metric.

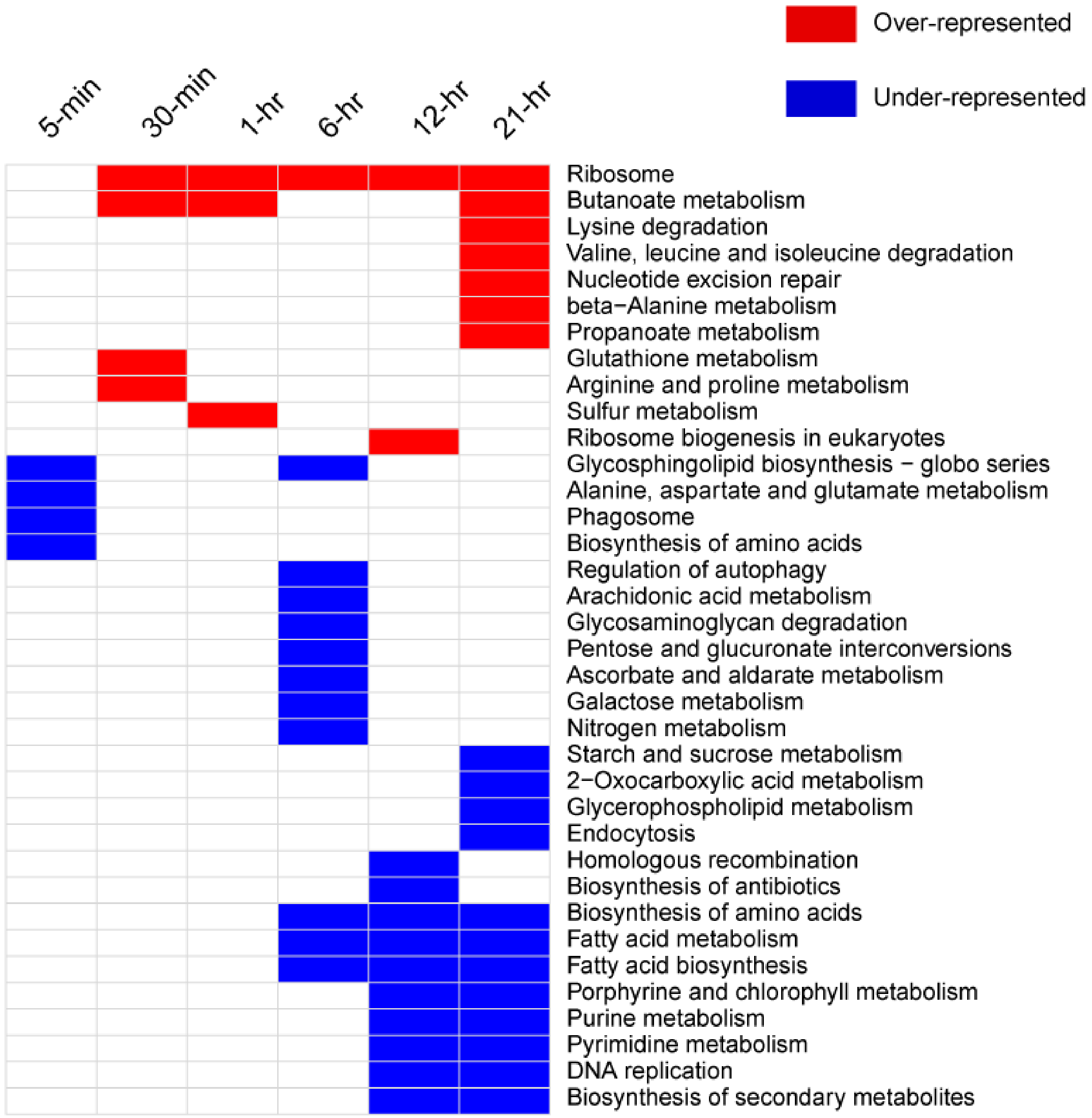

Supplementary Figure 6: Notable KEGG pathways over- or underrepresented in the virus treated samples across different time points. The overrepresented pathways are shown in orange, while the underrepresented ones are as blue rectangles.

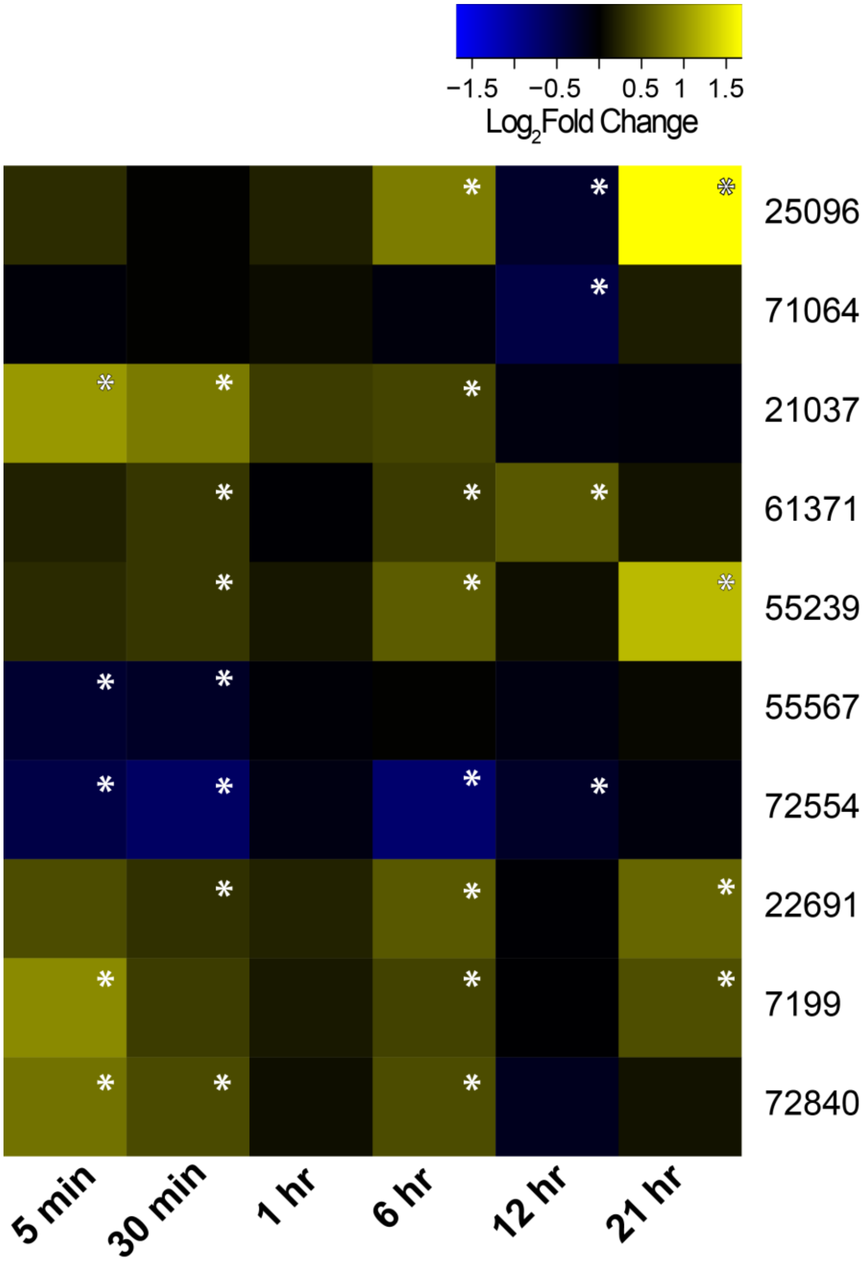

Supplementary Figure 7: Expression profile of genes involved in mismatch repair in the virus infected culture compared to healthy controls. Joint Genomic Institute (JGI) proteinIDs are presented on the right side of the heatmap. Annotated genes are as follows: 25096, 71064, 21037, 61371: MutS family proteins, 55239, 55567, 72554: MutL family proteins, 22691: Small MutS related (smr) protein, 7199, 72840: MutS V domain containing proteins.

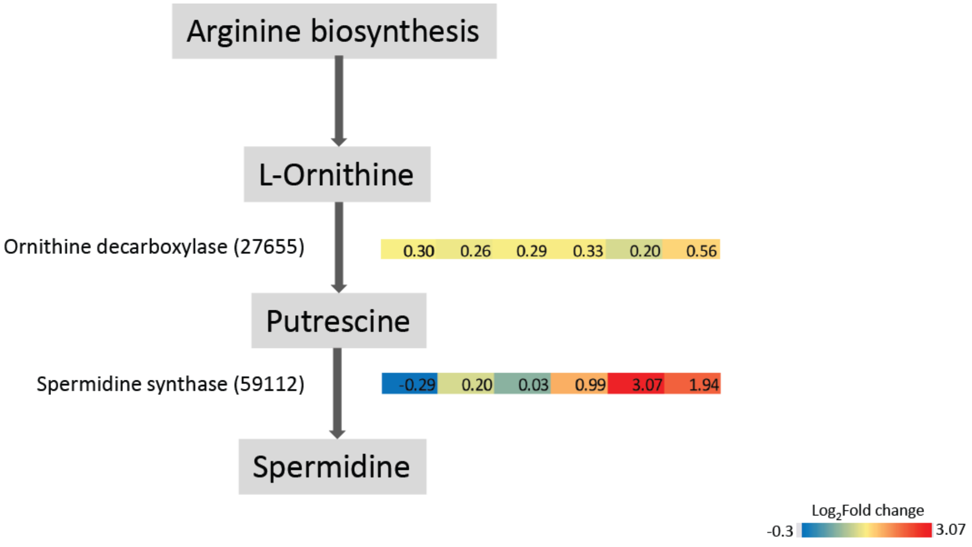

Supplementary Figure 8: Spermidine biosynthesis pathway in *Aureococcus*. The expression changes (Log2 fold change) compared to control are presented as heatmaps for each of the genes at right side of the pathway. The heatmaps represent fold changes over progressive sampling time from left to right. The JGI protein IDs are given in parentheses along with the gene names.

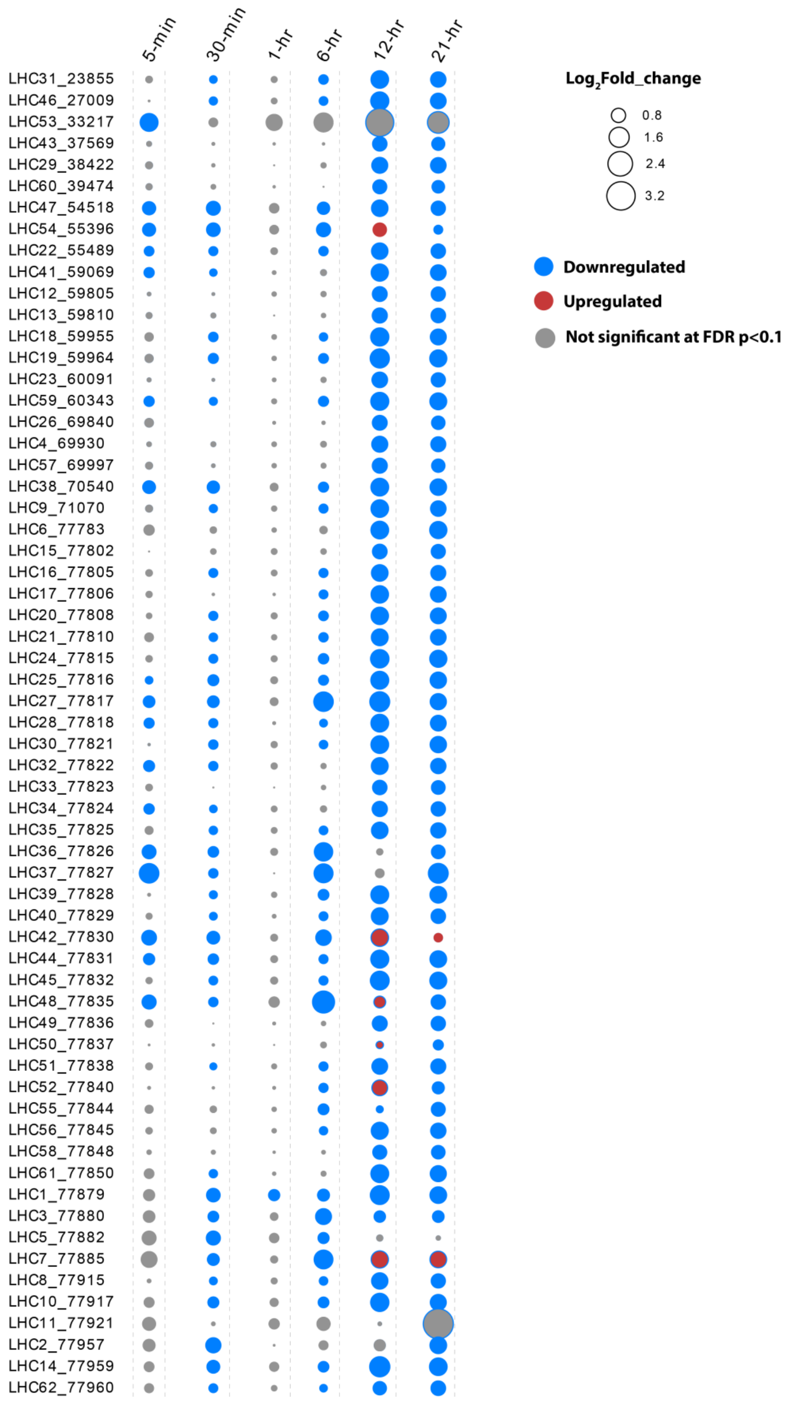

Supplementary Figure 9: The expression pattern of all the light harvesting complex (LHC) protein encoding genes in virus treated *Aureococcus* cultures compared to the control. Fold changes are Log2 converted. Positive fold changes are in red, while negative fold changes are shown in blue. Non-significant (FDR p>0.1) fold changes are shown in gray. The Joint Genomic Institute (JGI) protein IDs for the genes are provided on left in the format: 'LHCxx_JGI ID'

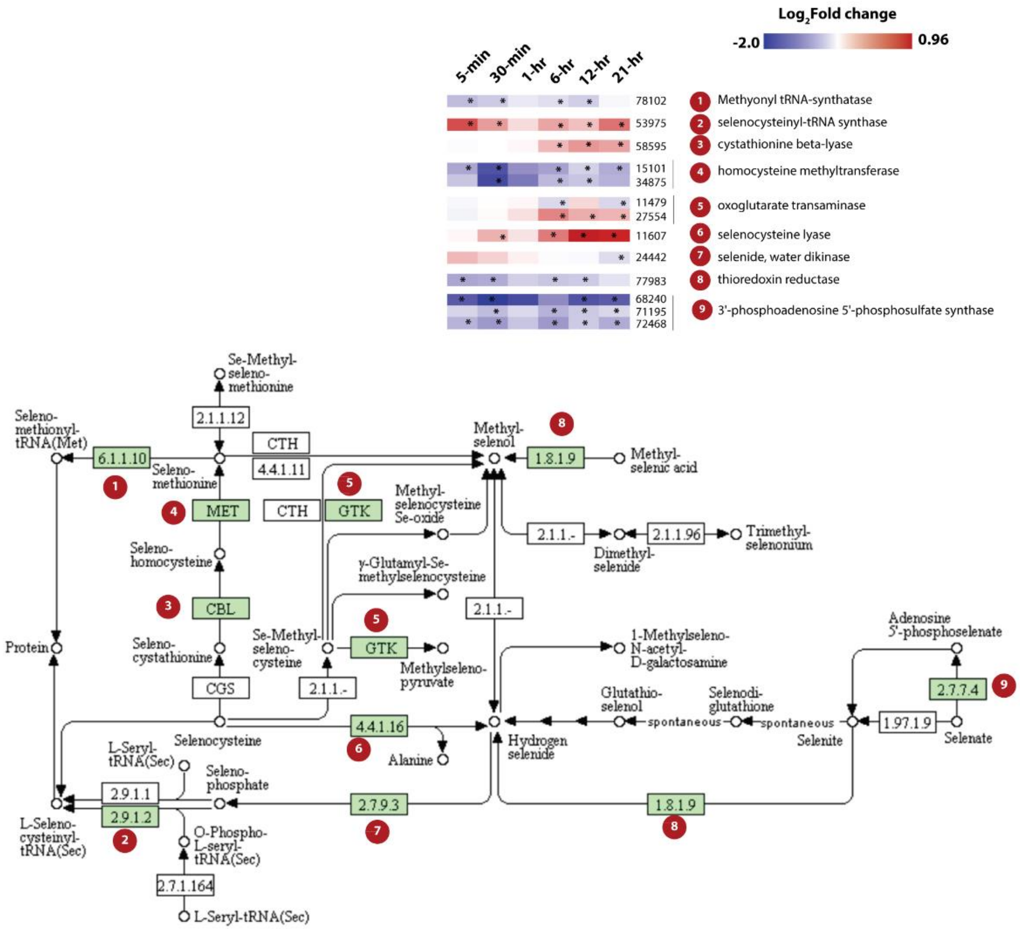

Supplementary Figure 10: KEGG pathway “Selenocompound metabolism” with *Aureococcus* gene mapped on it. Enzyme coding genes having homologs in *Aureococcus* genome are denoted in green. The expression pattern of these genes (Log2 fold change) compared to the control are presented in the heatmap. Significant (FDR p<0.1) fold changes are marked with asterisk.

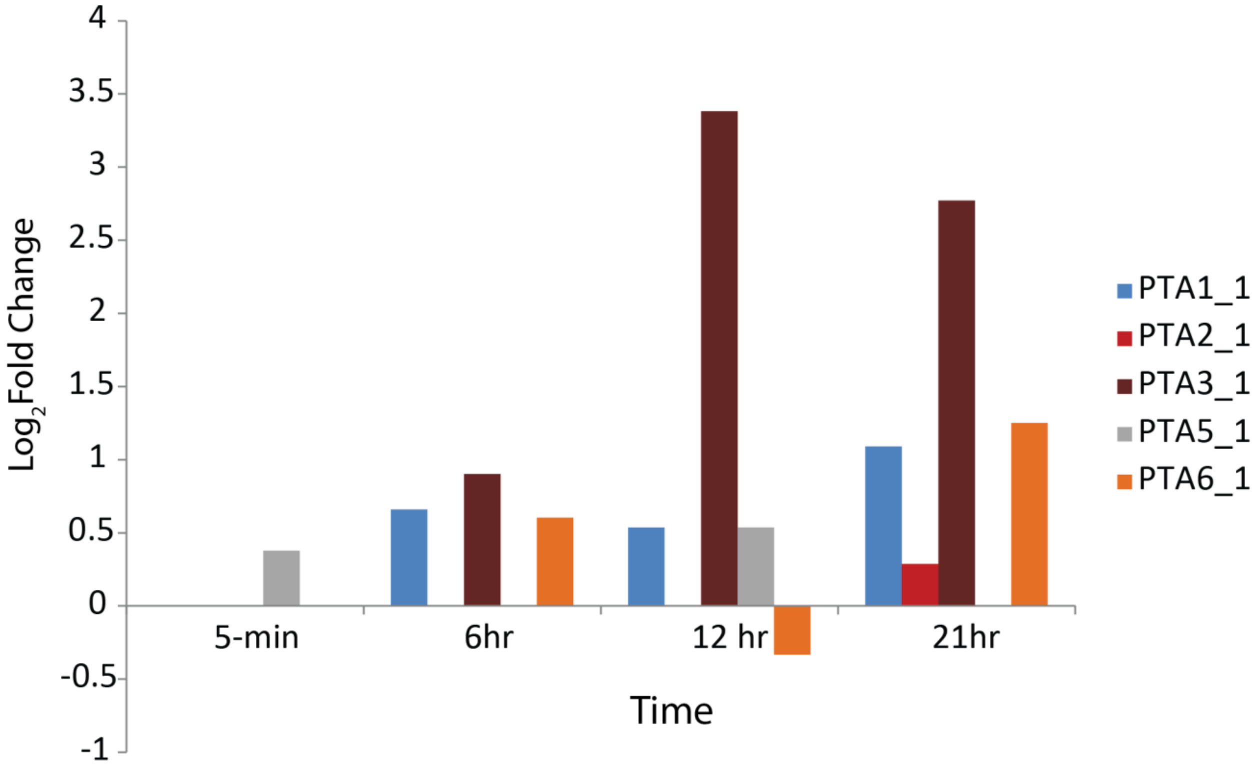

Supplementary Figure 11: Up- or down-regulation of annotated phosphate transporters in *Aureococcus* during 5 min, 6 hr, 12 hr and 21 hr post-infection. No significant expression change (FDR corrected p≤0.1) was detected for these genes during 30 min and 1 hr post-infection.

Supplementary Table 1: Number of reads mapped from each sequence library on host (nucleus, mitochondria, chloroplast) and virus genomes. Unmapped reads refer to the number of reads that could not be mapped at a 95% similarity and 70% length matching criteria.

		**Control samples**					**Virus treated samples**					
**Time**	**replicates**	Trimmed library size	nuclear	mitochondrial	chloroplast	unmapped	Trimmed library size	nuclear	mitochondrial	chloroplast	virus	unmapped
5 min	A	25,429,557	19,759,179	12,872	101,974	5,555,339	21,319,597	16,308,486	7,956	71,740	2,839	4,928,576
	B	25,178,005	19,741,166	7,700	69,481	5,359,520	25,210,205	19,278,462	11,902	100,183	745	5,818,913
	C	25,147,873	19,518,862	11,374	85,146	5,532,405	23,597,141	18,215,228	13,585	115,291	1,162	5,251,875
30 min	A	23,781,080	18,620,949	5,474	58,307	5,096,200	22,778,212	17,720,061	6,838	73,628	4,399	4,973,286
	B	25,065,355	19,319,869	6,892	65,798	5,672,667	25,051,680	19,541,721	11,610	105,749	3,568	5,389,032
	C	22,544,877	17,623,936	5,358	52,409	4,863,102	24,355,117	18,874,147	12,761	101,418	3,716	5,363,075
1 hr	A	22,140,838	17,518,145	3,622	35,487	4,583,442	24,866,410	19,570,495	7,647	87,749	12,899	5,187,620
	B	22,816,003	17,702,432	7,799	65,618	5,039,961	22,878,269	18,036,620	6,774	58,841	10,896	4,765,138
	C	24,204,118	18,623,392	10,169	95,699	5,474,348	24,765,001	19,023,306	12,391	93,598	15,403	5,620,303
6 hr	A	29,412,598	22,885,763	8,010	108,002	6,410,532	18,164,525	13,987,866	8,980	68,385	187,950	3,911,344
	B	29,353,924	23,000,533	7,806	73,858	6,271,221	23,383,040	18,238,144	9,897	78,915	231,715	4,824,369
	C	20,514,018	16,132,599	6,235	46,560	4,328,501	26,135,590	19,960,379	16,760	119,043	284,571	5,754,837
12 hr	A	26,757,048	21,376,546	3,269	40,636	5,336,429	24,082,190	18,831,228	6,108	54,959	460,209	4,729,686
	B	24,291,501	19,385,000	3,231	39,028	4,864,019	25,560,037	19,864,499	8,871	63,887	526,936	5,095,844
	C	22,918,888	18,314,454	4,332	44,947	4,555,031	27,406,879	20,803,231	11,548	93,999	636,383	5,861,718
21 hr	A	24,580,272	19,167,142	6,741	74,405	5,331,088	26,996,441	17,412,549	14,001	90,734	3,833,543	5,645,614
	B	25,439,000	19,013,752	9,064	87,314	6,328,543	27,294,047	17,041,117	17,372	104,429	3,977,800	6,153,329
	C	23,206,823	17,925,810	5,225	59,616	5,216,055	24,678,583	15,215,726	13,859	88,983	3,862,182	5,497,833

Supplementary table 2: AaV genes with COG/NCVOG assignments and time point when their expressions were first observed. Genes with no COG/NCVOG assignments are not shown. A number of genes were possibly horizontally transferred and unique to AaV among the NCLDVs (Moniruzzaman et al, 2014). Names of these genes are marked with '**' and their putative phylogenetic origins are shown within parentheses.

Gene name	Annotation	COG category	NCVOG category	Time of first expression
AaV_320	DNA directed RNA polymerase largest subunit	0086 (Transcription)	0274 (Transcription and RNA processing)	5 min
AaV_193	DNA directed RNA polymerase II subunit rpb3	0202 (Transcription)	0635 (RNA_polymerase,Transcription and RNA processing)	5 min
AaV_124	NAD dependent DNA ligase	0272 (DNA replication, recombination & repair)	0035 (DNA replication, recombination & repair)	5 min
AaV_042	Putative Lon protease	0466 (Posttranslational modification, protein turnover, chaperones)	0228 (Other metabolic functions)	5 min
AaV_084	Topoisomerase Type IA	0550 (DNA replication, recombination & repair)	0033 (DNA replication, recombination & repair)	5 min
AaV_088	Putative DNA topoisomerase IA	0550 (DNA replication, recombination & repair)	0036 (DNA replication, recombination & repair)	5 min
AaV_235	Putative Helicase/ E3 Ubiquitin Ligase	0553 (Transcription/DNA replication,recombination,repair)	0330 (Signal transduction regulation)	5 min
AaV_318	Putative deoxyuridine 5'_triphosphate nucleotidohydrolase (dUTPase)	0756 (Nucleotide transport and metabolism)	1068 (Nucleotide metabolism)	5 min
AaV_306	Putative ABC-transporter family protein	1132 (Defense mechanisms)	0002 (Miscellaneous)	5 min
AaV_203	Transcription factor TF IIB	1405 (Transcription)	1127 (Transcription and RNA processing)	5 min
AaV_381	Transcription Elongation factor TFIIS/DNA directed RNA polymerase subunit M	1594 (Transcription)	0272 (Transcription and RNA processing)	5 min

Gene name	Annotation	COG category	NCVOG category	Time of first expression
AaV_308	Putative bZIP transcription factor	1792 (Cell envelope biogenesis, outer membrane)	-	5 min
AaV_375**	Putative pectate lyase (Bacteria/*Aureococcus)*	3866 (Carbohydrate transport and metabolism)	-	5 min
AaV_078**	Family 88 Glycosyl Hydrolase (Unsaturated Glucuronyl hydrolase) (*Aureococcus*)	4225 (General function prediction only)	-	5 min
AaV_066	SUMO-1 specific cysteine protease	5160 (Posttranslational modification, protein turnover, chaperones)	0246 (Other metabolic functions)	5 min
AaV_380	Putative Zinc finger domain containing protein	5189 (Transcription / Cell division and chromosome partitioning)	0072 (DNA replication, recombination & repair)	5 min
AaV_118	Translation elongation factor EF-1 alpha	5256 (Translation, ribosomal structure and biogenesis)	0064 (Translation)	5 min
AaV_075	Class I DNA binding Protein	5648 (Chromatin structure and dynamics)	0071 (Miscellaneous)	5 min
AaV_274	Beta-1,4 Galactosyltransferase	-	0044 (Other metabolic functions)	5 min
AaV_228	C3HC4 domain containing E3 ubiquitin ligase	-	0330 (Signal transduction regulation)	5 min
AaV_292	C3HC4 type E3 Ubiquitin ligase	-	0330 (Signal transduction regulation)	5 min
AaV_151	Hypothetical protein	-	1278 (Uncharacterized)	5 min
AaV_246	Hypothetical protein	-	0632 (Uncharacterized)	5 min
AaV_179	Hypothetical protein	-	1137 (Uncharacterized)	5 min
AaV_211	mRNA capping Enzyme	-	1117 (Transcription and RNA processing)	5 min
AaV_269	Putative ATP-dependent RNA helicase	-	0031 (DNA replication, recombination & repair)	5 min
AaV_096	Putative Capsid protein	-	0022 (Virion structure and morphogenesis)	5 min

Gene name	Annotation	COG category	NCVOG category	Time of first expression
AaV_316	Putative CMP/dCMP deaminase	-	1064 (Other metabolic functions)	5 min
AaV_024	Putative Concanavalin A-like lectin/glucanase superfamily protein	-	0107 (Uncharacterized)	5 min
AaV_329	Putative DNA polymerase III subunit alpha (partial)	-	0420 (Uncharacterized)	5 min
AaV_097	Putative HNH endonuclease	-	0072 (DNA replication, recombination & repair)	5 min
AaV_159	Putative lambda-type exonuclease	-	1192 (DNA replication, recombination & repair)	5 min
AaV_117	Putative TATA-box binding family protein.	-	0313 (DNA replication, recombination & repair)	5 min
AaV_361	Putative VLTF-3 like Transcription Factor	-	0262 (Transcription and RNA processing)	5 min
AaV_112	Type II DNA modification methyl transferase	-	0234 (Other metabolic functions)	5 min
AaV_141	B family DNA polymerase	0417 (DNA replication, recombination & repair)	0038 (DNA replication, recombination & repair)	30 min
AaV_192	Putative glycosyltransferase	0438 (Cell envelope biogenesis, outer membrane)	0067 (Other metabolic functions)	30 min
AaV_043**	Small conductance mechanosensetive channel (Ambiguous)	0668 (Cell envelope biogenesis, outer membrane)	-	30 min
AaV_368	Putative Histone acetyl transferase	1243 (Transcription/Chromatin structure and dynamics)	-	30 min
AaV_288	Putative SAM dependent methyltransferase	2227 (Coenzyme metabolism)	1191 (Other metabolic functions)	30 min
AaV_175	Conserved hypothetical protein	-	1216 (Uncharacterized)	30 min
AaV_142	DnaJ/Heat shock protein 40	-	0046 (Miscellaneous)	30 min

Gene name	Annotation	COG category	NCVOG category	Time of first expression
AaV_214	Hypothetical protein	-	0645 (Uncharacterized)	30 min
AaV_249	Hypothetical protein	-	1343 (Miscellaneous)	30 min
AaV_153	Putative Ion channel domain containing protein	-	1344 (Other metabolic functions)	30 min
AaV_134	Putative VLTF2 like transcription factor	-	1164 (Transcription and RNA processing)	30 min
AaV_293	DNA topoisomerase type IIA	0187 (DNA replication, recombination & repair)	0037 (DNA replication, recombination & repair)	1 hr
AaV_054	Thymidylate synthase	0207 (Nucleotide transport and metabolism)	1136(Nucleotide metabolism)	1 hr
AaV_109	Ribonucleoside di phosphate reductase alpha subunit	0209 (Nucleotide transport and metabolism)	1353 (Nucleotide metabolism)	1 hr
AaV_208	Protein Disulfide isomerase	0526 (Posttranslational modification, protein turnover, chaperones)	0317 (Other metabolic functions)	1 hr
AaV_079	Putative Exoribonuclease (Ribonuclease R)	0557 (Transcription)	-	1 hr
AaV_077**	Rhomboid family Serine protease (*Aureococcus)*	0705 (Amino acid metabolism)	-	1 hr
AaV_324	Putative type II DNA methyltransferase	2263 (Translation, ribosomal structure and biogenesis)	0234 (Other metabolic functions)	1 hr
AaV_357	SCF ubiquitin ligase	5201 (Posttranslational modification, protein turnover, chaperones)	1299 (Other metabolic functions)	1 hr
AaV_232	Hypothetical protein	-	1131 (Uncharacterized)	1 hr
AaV_200	Hypothetical protein	-	1423 (Uncharacterized)	1 hr
AaV_113	Putative adenine specific DNA methyltransferase	-	0234 (Other metabolic functions	1 hr
AaV_226	Putative D5 Primase/Helicase	-	0023 (DNA replication, recombination & repair)	1 hr

Gene name	Annotation	COG category	NCVOG category	Time of first expression
AaV_201	Putative Holliday junction resolvase	-	0278 (DNA replication, recombination & repair)	1 hr
AaV_242	DNA directed RNA polymerase II largest subunit	0086 (Transcription)	0274 (Transcription and RNA processing)	6 hr
AaV_244	Ribonulease HII	0164 (DNA replication, recombination & repair)	-	6 hr
AaV_171	DNA mismatch repair ATPase (MutS)	0249 (DNA replication, recombination & repair)	0105 (DNA replication, recombination & repair)	6 hr
AaV_322	Cytosine-C5 specific DNA methyl transferase	0270 (DNA replication, recombination & repair)	1066 (Nucleotide metabolism)	6 hr
AaV_295	Putative N6 Adenine specific DNA methyltransferase	0286 (Defense mechanisms)	0234 (Other metabolic functions)	6 hr
AaV_282	Putative DNA repairing ATPase	0419 (DNA replication, recombination & repair)	0308 (DNA replication, recombination & repair)	6 hr
AaV_234	Nucleoside diphosphate hydrolase (MutT)	0494 (DNA replication, recombination, & repair / General function prediction only)	0236 (Transcription and RNA processing)	6 hr
AaV_173	Nucleoside diphosphate hydrolase (MutT)	0494 (DNA replication, recombination, & repair / General function prediction only)	0236 (Transcription and RNA processing)	6 hr
AaV_076	RNA polymerase sigma factor 70	0568 (Transcription)	-	6 hr
AaV_065	Proliferating cell nuclear antigen (PCNA)/DNA polymerase sliding clamp	0592 (DNA replication, recombination & repair)	0241 (DNA replication, recombination & repair)	6 hr
AaV_071	Cyclophilin type peptydyl-prolyl cis-trans isomerase	0652 (Posttranslational modification, protein turnover, chaperones)	0711 (Other metabolic functions)	6 hr
AaV_128	DNA (cytosine-5) methyltransferase	0863 (DNA replication, recombination & repair)	-	6 hr
AaV_271	RNA polymerase subunit Rpb10	1644 (Transcription)	1368 (Transcription and RNA processing)	6 hr
AaV_044	DNA polymerase X family protein	1796 (DNA replication, recombination & repair)	004 (DNA replication, recombination & repair)	6 hr

Gene name	Annotation	COG category	NCVOG category	Time of first expression
AaV_094	Putative deoxycytidylate deaminase	2131 (Nucleotide transport and metabolism)	1064 (Other metabolic functions)	6 hr
AaV_372	Putative Phaeophorbide a Oxygenase	2146 (Inorganic ion transport and metabolism / General function prediction only)		6 hr
AaV_378	Glycosyl Transferase family 25	3306 (Cell envelope biogenesis, outer membrane)	1198 (Other metabolic functions)	6 hr
AaV_323	D5-ATPase-Helicase	3378 (General function prediction only)	0023 (DNA replication, recombination & repair)	6 hr
AaV_038	Putative Pectate Lyase	3866 (Carbohydrate transport and metabolism)	-	6 hr
AaV_099	Putative Polynucleotide-kinase-3 phosphatase	4088 (Nucleotide transport and metabolism)	0243 (Other metabolic functions)	6 hr
AaV_030	Putative Superfamily II RNA helicase	4581 (DNA replication, recombination & repair)	0030 (DNA replication, recombination & repair)	6 hr
AaV_174	DNA directed RNA polymerase K subunit/rpb6	-	0522 (Transcription and RNA processing)	6 hr
AaV_383	fucosylgalactoside 3-alpha-galactosyltransferase	-	0059 (Uncharacterized)	6 hr
AaV_126	Hypothetical protein	-	0842 (Uncharacterized)	6 hr
AaV_223	Hypothetical protein	-	1024 (Uncharacterized) (exclusive to iridoviruses, this virus also have this)	6 hr
AaV_186	Hypothetical protein	-	1129 (Uncharacterized)	6 hr
AaV_303	Oxogluterate/Iron dependent dioxygenase	-	1166 (Other metabolic functions)	6 hr
AaV_073	Putative DEADDEAh box helicase	-	0032 (DNA replication, recombination & repair)	6 hr
AaV_209	Putative thioredoxin like fold containing protein	-	0629 (Uncharacterized)	6 hr

Gene name	Annotation	COG category	NCVOG category	Time of first expression
AaV_255	Undecaprenyl pyrophosphate synthase	0020 (Lipid metabolism)	-	12 hr
AaV_222	RNA polymerase beta subunit	0085 (Transcription)	0271 (Transcription and RNA processing)	12 hr
AaV_132	Ribonucleotide reductase small subunit	0208 (Nucleotide transport and metabolism)	0276 (Nucleotide metabolism)	12 hr
AaV_110**	Eukaryotic Translation Elongation factor 5A *(Aureococcus)*	0231 (Translation, ribosomal structure and biogenesis)	-	12 hr
AaV_241	Ribonuclease H	0258 (DNA replication, recombination & repair)	-	12 hr
AaV_125	Putative ribonuclease H1	0328 (DNA replication, recombination & repair)	1352(DNA replication, recombination & repair)	12 hr
AaV_373**	UbiA Prenyltransferase (Ambiguous)	0382 (Coenzyme metabolism)	-	12 hr
AaV_093**	Putative Cytosine Deaminase (Bacteria)	0402 (Nucleotide transport and metabolism / General function prediction only)	-	12 hr
AaV_082	Putative DNA photolyase class II	0415 (DNA replication, recombination & repair)	1004 (Other metabolic functions)	12 hr
AaV_170	Putative Deoxynucleoside kinase	0480 (Translation, ribosomal structure and biogenesis)	1067 (Nucleoside/Nucleotide_kinase,Nucleotide metabolism)	12 hr
AaV_178	Serine threonine protein phosphatase	0639 (Signal transduction mechanisms)	0995 (Uncharacterized). Renized by deltablast	12 hr
AaV_367	Putative RNA methylase	0742 (DNA repilcation, recombination & repair)	0564 (Other metabolic functions)	12 hr
AaV_315	Putative DNA polymerase epsilon subunit	0847 (DNA replication, recombination & repair)	0047 (DNA replication, recombination & repair)	12 hr
AaV_180	Putative VV D6R-type Helicase	1061 (Transcription / DNA replication, recombination, & repair)	0031 (DNA replication, recombination & repair)	12 hr
AaV_130	VV_A18 like Helicase	1061 (Transcription/DNA replication,recombination,repair)	0076 (DNA replication, recombination & repair)	12 hr

Gene name	Annotation	COG category	NCVOG category	Time of first expression
AaV_261	Putative HD superfamily phosphohydrolase	1078 (General function prediction only)	0603 (Uncharacterized)	12 hr
AaV_311	Superfamily I Helicase	1112 (DNA replication, recombination & repair)	-	12 hr
AaV_210	Phosphate starvation-inducible protein PhoH	1702 (Signal transduction mechanisms)	-	12 hr
AaV_215	Crossover junction endonuclease Mus81	1948 (DNA replication, recombination & repair)	-	12 hr
AaV_224	RNA polymerase subunit RPB5	2012 (Transcription)	0273 (Transcription and RNA processing)	12 hr
AaV_287	S-adenosyl L-methionine dependent Methyltransferase	2226 (Coenzyme metabolism)	1191 (Other metabolic functions)	12 hr
AaV_034	Replication factor C subunit 2	2256 (DNA replication, recombination & repair)	1351 (DNA replication, recombination & repair)	12 hr
AaV_036	Oxogluterate/Iron dependent dioxygenase	3128 (Function unknown)	1166 (Other metabolic functions)	12 hr
AaV_359	family 25 Glycosyltransferase	3306(Cell envelope biogenesis, outer membrane)	0068 (Other metabolic functions)	12 hr
AaV_111	Class 3 Lipase	3675 (Lipid metabolism)	0225 (Other metabolic functions).	12 hr
AaV_003**	Putate pectate lyase *(Aureococcus/Bacteria)*	3866 (Carbohydrate transport and metabolism)	-	12 hr
AaV_290	ERV/ALR sulphydryl oxidase	5054 (Posttranslational modification, protein turnover, chaperones)	0052 (Virion structure and morphogenesis)	12 hr
AaV_152	C3H2C3-type E3 Ubiquitin ligase/RING-H2 finger protein	5194 (Posttranslational modification, protein turnover, chaperones)	0330 (Signal transduction and regulation)	12 hr
AaV_298	DNA directed RNA polymerase subunit rpb9/M	-	0521 (Uncharacterized)	12 hr
AaV_307	DnaJ/Heat shock protein 40	-	0046 (Miscellaneous)	12 hr

Gene name	Annotation	COG category	NCVOG category	Time of first expression
AaV_250	Hypothetical protein	-	0628 (Uncharacterized)	12 hr
AaV_328	Hypothetical protein	-	1083 (Uncharacterized)	12 hr
AaV_243	Hypothetical protein	-	1278 (Uncharacterized)	12 hr
AaV_239	Hypothetical protein	-	1343 (Miscellaneous)	12 hr
AaV_116	Hypothetical protein	-	0158 (Uncharacterized)	12 hr
AaV_074	Hypothetical protein	-	0329 (Other metabolic functions)	12 hr
AaV_330	Hypothetical protein	-	1012 (Uncharacterized)	12 hr
AaV_265	Hypothetical protein	-	1343 (Miscellaneous)	12 hr
AaV_285	Hypothetical protein	-	1343 (Miscellaneous)	12 hr
AaV_247	Putative Capsid protein 2	-	0022 (Virion structure and morphogenesis)	12 hr
AaV_386	Putative Concanavalin A-like lectin/glucanase superfamily protein	-	0108 (Uncharacterized)	12 hr
AaV_158	Putative metal dependent hydrolase	-	1120 (Metallopeptidase)	12 hr
AaV_190	Putative Serine threonine protein kinase haspin	-	0285 (Signal transduction regulation).	12 hr
AaV_233	Replication factor C small subunit 2	-	0001 (DNA replication, recombination & repair)	12 hr
AaV_227	Replication factor C subunit	-	0071 (Miscellaneous)	12 hr
AaV_002	RNA polymerase II second largest subunit	0085 (Transcription)	0271 (Transcription and RNA processing)	21 hr
AaV_253	RNA polymerase Rpb5, C-terminal domain	2012 (Transcription)	0273 (Transcription and RNA processing)	21 hr
AaV_123	S-phase-kinase-associated protein-1 (Skp1)	5201 (Posttranslational modification, protein turnover, chaperones)	1299 (Other metabolic functions)	21 hr

Gene name	Annotation	COG category	NCVOG category	Time of first expression
AaV_165	Putative A32 Virion packaging ATPase	-	0249 (Virion structure and morphogenesis)	21 hr

References for Supplementary table 2:

Moniruzzaman, M., Lecleir, G.R., Brown, C.M., Gobler, C.J., Bidle, K.D., and Wilson, W.H. (2014). Genome of brown tide virus (AaV), the little giant of the Megaviridae, elucidates NCLDV genome expansion and host–virus coevolution. *Virology*. 466-467,60-70.

Supplementary Table 3: Expression profile of *Aureococcus* guanylate binding proteins (GBP) in the infected cultures compared to control. Significant fold changes (FDR p<0.1) are marked in blue. Protein ID – JGI protein identification numbers, FDR_P – FDR corrected P-values, FC – Fold change.

	5 minutes		30 minutes		1 hour		6 hours		12 hours		21 hours	
Protein_ID	FC	FDR_P	FC	FDR_P	FC	FDR_P	FC	FDR_P	FC	FDR_P	FC	FDR_P
19411	-1.19	0.75	-1.69	1.77E-03	-1.26	1	-1.88	5.14E-04	-1.34	0.02	-1.24	0.35
20854	1.09	1	-1.36	0.22	1	1	-1.61	0.02	-1.69	2.95E-06	-1.15	0.55
22961	-1.05	1	-1.13	1	1.55	1	1.06	1	-1.06	1	-4.14	0.4
23822	1.12	1	-1.05	1	-1.52	1	1.3	0.72	1.14	0.97	-1.03	1
29376	-1.01	1	-1.44	6.72E-03	-1.05	1	-1.32	0.14	1.12	0.29	1.28	0.1
9916	1.14	0.78	-1.1	0.7	-1.19	1	-1.54	9.50E-04	-1.91	2.94E-07	-1.22	0.15
70671	-1.29	0.19	-1.45	1.00E-05	-1.22	0.95	-1.7	1.01E-09	-1.97	3.07E-15	-2.24	0

Supplementary Table 4: Over and underexpressed genes encoding components of photosystem I and II across the time course compared to uninfected control. Fold changes (FC) and corresponding FDR p values are shown. Significant (FDR p<0.1) positive (orange) and negative (green) fold changes are marked.

	5-min		30-min		1-h		6-h		12-h		21-h	
Gene	FC	FDR p	FC	FDR p	FC	FDR p	FC	FDR p	FC	FDR p	FC	FDR p
psaA	-1.84	0.00	-1.58	0.00	-1.54	0.05	-1.89	0.00	-1.43	0.00	-1.74	0.00
psaB	-1.74	0.00	-1.30	0.05	-1.47	0.65	-1.28	0.11	1.03	1.00	-1.20	0.59
psaC	-1.06	1.00	-1.91	0.30	-1.80	1.00	1.25	0.74	1.62	0.36	-2.21	0.06
psaD	-1.41	0.88	-1.18	1.00	-1.83	0.86	1.16	0.96	1.28	0.51	-1.20	0.97
psaJ	1.64	1.00	-2.63	0.74	-6.73	1.00	-1.73	0.96	-1.82	1.00	2.31	0.85
psaL	-2.34	0.00	-1.50	0.09	-1.38	1.00	-1.82	0.00	-1.14	0.41	-1.06	0.99
psaM	1.00	1.00	-2.81	0.91	-6.72	1.00	1.07	1.00	-1.82	1.00	1.00	1.00
psbA	-1.31	0.09	-1.46	0.00	-1.56	0.17	-1.07	0.79	1.57	0.00	1.51	0.04
psbB	1.11	0.88	1.68	0.00	-1.02	1.00	1.52	0.00	1.37	0.01	-1.04	0.90
psbC	-1.61	0.00	-1.26	0.08	-1.65	0.11	-1.06	0.93	1.07	0.83	1.08	0.93
psbD	-1.85	0.00	-1.60	0.00	-1.56	0.36	-1.58	0.00	1.24	0.06	1.29	0.06
psbE	-1.37	0.88	-1.11	1.00	-1.21	1.00	-1.40	0.44	1.13	1.00	-1.16	1.00

psbF	-1.33	1.00	-2.63	0.75	-1.14	1.00	-1.50	0.90	2.88	0.22	-2.42	0.70
psbH	-1.57	0.96	-3.14	0.02	-1.78	1.00	-1.23	1.00	-1.07	1.00	-1.55	0.45
psbI	1.25	0.46	1.73	0.09	-1.37	1.00	-1.36	0.10	-1.23	0.40	-1.14	0.77
psbJ	-1.31	1.00	1.23	1.00	-2.08	1.00	-1.11	1.00	2.13	0.70	2.31	0.85
psbK	1.16	1.00	-1.17	1.00	-1.56	1.00	-1.04	1.00	1.03	1.00	4.47	0.07
psbL	-1.38	1.00	-1.16	1.00	-3.05	1.00	-1.58	0.83	2.93	0.34	-3.39	0.61
psbN	-3.76	1.00	-1.97	1.00	1.00	1.00	1.06	1.00	-6.52	0.74	3.57	1.00
psbT	-1.35	0.92	-1.29	0.95	-1.13	1.00	1.07	1.00	1.16	1.00	1.44	0.83
psbV	-1.47	0.65	-1.38	0.60	-1.52	1.00	-1.99	0.05	1.40	0.41	1.68	0.26
psbX	-1.05	1.00	1.62	0.79	3.50	1.00	-1.31	0.91	3.70	0.39	-1.06	1.00

Supplementary table 5: Expression profile of isoprenoid biosynthesis genes across the time points in the infected culture compared to healthy controls. Gene annotations are as follows: 23670: putative 4-diphosphocytidyl-2C-methyl-D-erythritol, 27565: isopentenyl pyrophosphate:dimethylallyl pyrophosphate isomerase, 32168: solanesyl diphosphate synthase, 36976: polyprenyl synthetase, 52464: geranylgeranyl diphosphate synthase, 55083: solanesyl diphosphate synthase, 59216: farnesyl pyrophosphate synthetase, 59945: polyprenyl synthetase, 70598: acetyl coenzyme-A synthetase, 71238: octaprenyl diphosphate synthetase, 72299: tetracorticopeptide domain containing protein, 71962: replication factor -A protein 1 (ssDNA binding protein p68 subunit), 2913: 1-deoxy D-xylulose 5-phosphate reductoisomerase, 4868: prenyl cysteine carboxyl methyltransferase.

	5 minutes		30 minutes		1 hour		6 hour		12 hour		21 hour	
JGI ID	FC	FDR p	FC	FDR p	FC	FDR p	FC	FDR p	FC	FDR p	FC	FDR p
23670	1.25	0.18	1.1	0.46	-1.05	1	-1.12	0.46	-1.27	0.48	-1.52	7.47E-08
27565	-1.37	0.36	-2.08	8.76E-09	-1.34	1	-1.95	9.49E-07	-1.62	3.44E-04	-1.52	8.41E-03
2913	-1.04	1	-1.24	4.43E-03	-1.19	0.92	-1.37	3.63E-05	-1.65	8.69E-13	-1.6	3.00E-11
32168	-1.05	1	-3.91	1	-1.1	1	1	1	-3.76	1	-1.83	1
36976	-1.76	6.44E-08	-1.21	8.47E-03	-1.15	1	-1.45	1.28E-06	-1.66	3.65E-14	-1.6	9.10E-10
4868	-1.35	0.33	-1.02	1	-1.1	1	-1.13	0.45	-1.2	0.08	1.36	0.07
52464	1.09	0.76	-1.21	8.24E-03	-1.19	0.9	-1.31	1.74E-04	-1.74	0	-1.66	1.06E-12
55083	-1.54	0.01	-1.76	2.13E-06	-1.17	1	-1.93	1.54E-04	-1.27	2.38E-03	-1.2	0.18
59216	-1.16	0.4	-1.16	0.09	-1.09	1	-1.25	6.77E-03	-1.59	3.49E-10	-1.1	0.34
59945	-1.37	9.69E-03	1.08	0.46	1.08	1	1.01	1	1.47	7.92E-10	1.36	5.77E-05

70598	1.45	0.02	-1.26	2.98E-03	-1.08	1	-1.37	9.17E-05	-1.9	0	-1.52	2.11E-08
71238	-1.18	0.3	-1.18	0.03	-1.18	0.93	-1.2	0.03	1.09	0.26	-1.1	0.41
71962	1.02	1	-1.11	0.25	1	1	1.05	0.76	-1.21	3.19E-03	1.37	2.80E-05
72299	-1.7	1	-3.12	0.56	-1.38	1	1.45	1	-14.72	0.15	-1.07	1
